# Mapping exclusive breastfeeding in Africa between 2000 and 2017

**DOI:** 10.1038/s41591-019-0525-0

**Published:** 2019-07-22

**Authors:** Natalia V. Bhattacharjee, Lauren E. Schaeffer, Laurie B. Marczak, Jennifer M. Ross, Scott J. Swartz, James Albright, William M. Gardner, Chloe Shields, Amber Sligar, Megan F. Schipp, Brandon V. Pickering, Nathaniel J. Henry, Kimberly B. Johnson, Celia Louie, Michael A. Cork, Krista M. Steuben, Alice Lazzar-Atwood, Dan Lu, Damaris K. Kinyoki, Aaron Osgood-Zimmerman, Lucas Earl, Jonathan F. Mosser, Aniruddha Deshpande, Roy Burstein, Lauren P. Woyczynski, Katherine F. Wilson, John D. VanderHeide, Kirsten E. Wiens, Robert C. Reiner, Ellen G. Piwoz, Rahul Rawat, Benn Sartorius, Nicole Davis Weaver, Molly R. Nixon, David L. Smith, Nicholas J. Kassebaum, Emmanuela Gakidou, Stephen S. Lim, Ali H. Mokdad, Christopher J. L. Murray, Laura Dwyer-Lindgren, Simon I. Hay

**Affiliations:** 1 Institute for Health Metrics and Evaluation, University of Washington, Seattle, WA USA; 2 Department of Global Health, University of Washington, Seattle, WA USA; 3 Department of Medicine, University of Washington, Seattle, WA USA; 4 Department of Health Metrics Sciences, University of Washington, Seattle, WA USA; 5 Bill & Melinda Gates Foundation, Seattle, WA USA; 6 Faculty of Infectious and Tropical Diseases, London School of Hygiene & Tropical Medicine, London, UK; 7 Department of Anesthesiology and Pain Medicine, University of Washington, Seattle, WA USA

**Keywords:** Public health, Risk factors, Paediatrics

## Abstract

Exclusive breastfeeding (EBF)—giving infants only breast-milk (and medications, oral rehydration salts and vitamins as needed) with no additional food or drink for their first six months of life—is one of the most effective strategies for preventing child mortality^1–4^. Despite these advantages, only 37% of infants under 6 months of age in Africa were exclusively breastfed in 2017^5^, and the practice of EBF varies by population. Here, we present a fine-scale geospatial analysis of EBF prevalence and trends in 49 African countries from 2000–2017, providing policy-relevant administrative- and national-level estimates. Previous national-level analyses found that most countries will not meet the World Health Organization’s Global Nutrition Target of 50% EBF prevalence by 2025^6^. Our analyses show that even fewer will achieve this ambition in all subnational areas. Our estimates provide the ability to visualize subnational EBF variability and identify populations in need of additional breastfeeding support.

## Abstract (web summary)

Exclusive breastfeeding in Africa is highly varied within and between countries, with many countries unlikely to reach World Health Organization 2025 targets without urgent action.

## Main

**Fig. 1:**
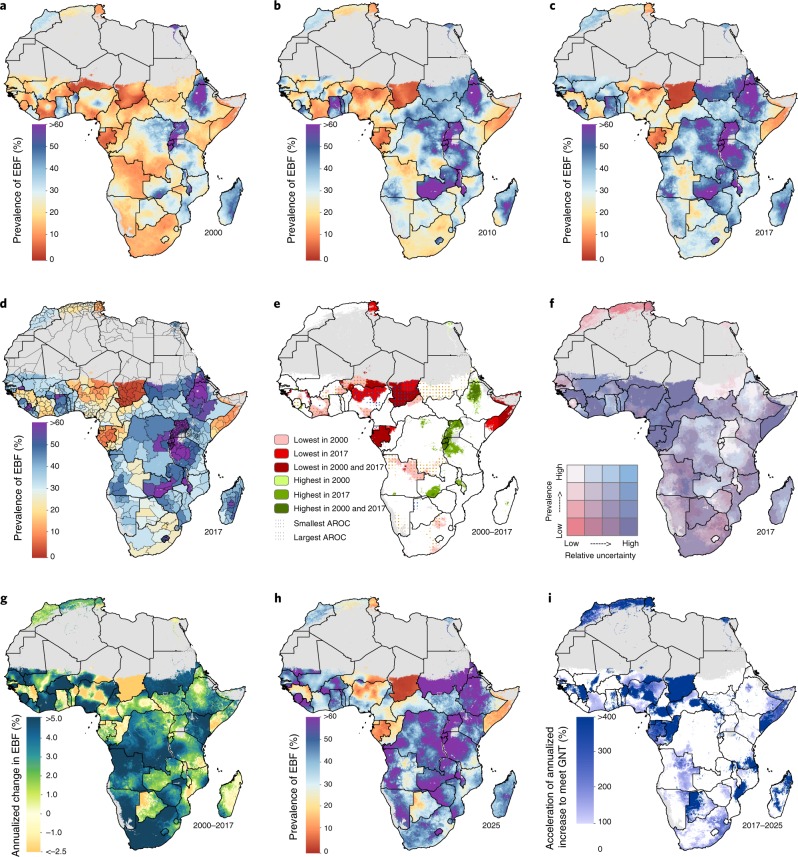
EBF prevalence (2000–2017) among infants under 6 months and progress towards the 2025 WHO GNT. **a**–**c**, Prevalence of EBF practices at the 5 km × 5 km resolution in 2000 (**a**), 2010 (**b**) and 2017 (**c**). **d**, Prevalence of EBF at the first administrative subdivision in 2017. **e**, Overlapping population-weighted lowest and highest 10% of grid cells and weighted AROC in EBF from 2000–2017. **f**, Overlapping population-weighted quartiles of EBF and relative 95% uncertainty in 2017. Cut-offs for the quartiles were 25.0% (25th percentile), 38.5% (50th percentile) and 52.3% (75th percentile) for the EBF prevalence axis, and 0.500 (25th percentile), 0.902 (50th percentile) and 0.137 (75th percentile) for the relative uncertainty axis (calculated as the absolute range of the uncertainty intervals divided by the estimate). **g**, Weighted annualized percentage change in EBF prevalence from 2000–2017. **h**, Grid cell level prevalence of EBF predicted for 2025, projected from 2017 based on AROC between 2000 and 2017. **i**, Acceleration in the annualized increase in EBF required to meet WHO GNT by 2025. Dark blue pixels were either non-increasing or must accelerate their rate of increase by more than 400% above 2000–2017 rates during 2017–2025 to achieve the target. White pixels require no increase to meet WHO GNT by 2025. Maps reflect administrative boundaries, land cover, lakes and population; gray-colored grid cells had fewer than ten people per 1 km × 1 km grid cell and were classified as ‘barren or sparsely vegetated’, or were not included in this analysis.

Previous national-level exclusive breastfeeding (EBF) prevalence estimates within Africa^4,7,8^ found substantial heterogeneity between countries, while studies comparing urban and rural locations^8,9^, and subnational-level estimates in select countries^8,10,11^, also identified considerable within-country heterogeneity. We found that EBF prevalence and trends varied greatly across the African continent between 2000 and 2017, often irrespective of national or subnational boundaries (Fig. 1a–d). The greatest observable patterns of improvement, where estimated EBF levels had increased from <25% to ≥40% in the modeled period, were along or near the East African Rift, including Sudan, South Sudan, Democratic Republic of the Congo (DRC), Kenya, Tanzania, Zambia and Malawi. Within these countries, an estimated 68 second administrative subdivisions (out of 534) had low EBF prevalence (estimates: <25%) in 2000, which subsequently increased to meet or exceed the World Health Organization’s (WHO’s) Global Nutrition Target (GNT; estimated EBF prevalence of ≥50%) by 2017. The estimated national prevalence nearly doubled in some countries in western (for example, Burkina Faso) and southern (for example, Namibia) sub-Saharan Africa (SSA) between 2000 and 2017. This was achieved by reducing the number of areas with low EBF prevalence. At the same time, estimates at higher spatial resolutions highlight corners of persistent need in countries that made notable national progress towards EBF targets, including in eastern Angola and eastern and coastal areas in South Africa. For example, we estimated a 13.6 percentage-point increase (95% uncertainty interval: 8.3–19.6) in national EBF prevalence in South Africa, from 10.2% (8.1–12.6%) in 2000 to 23.8% (18.5–30.0%) in 2017. Yet, areas with persistently lower levels, such as the City of Johannesburg (4.9% (2.9–7.7%) in 2000; 17.4% (10.4–27.0%) in 2017) and throughout Gauteng province (5.7% (3.5–8.7%) in 2000; 19.4% (12.0–29.0%) in 2017), contribute to South Africa’s relatively low national average.

Figure 1e features the best- and worst-performing locales by overlaying the highest- and lowest-prevalence areas (90th and 10th percentiles, respectively) across Africa in 2000 and 2017, and areas with the highest and lowest weighted annualized rates of change (AROC) for the 18-year study period. Burundi and Rwanda—nearly homogeneously—were among the top achievers in Africa in both 2000 and 2017, as were north-western parts of Ethiopia, areas scattered throughout Uganda, and south-western Zambia. Sudan showed some of the highest and most consistent rates of increase within its borders. Areas in southern Côte d’Ivoire, eastern and western Burkina Faso, south-western Niger, southern Nigeria, northern Central African Republic (CAR), northern Angola, southern DRC and central South Africa had among the lowest EBF prevalence in 2000; however, high AROC propelled most of these areas out of the lowest decile by 2017. Conversely, areas in northern Nigeria and throughout Chad were among the lowest-prevalence and lowest-AROC areas, indicating stagnation or reversals in progress. The majority of areas in Gabon and Somalia, as well as a large geographic area in south-eastern Niger and pockets in north-eastern Angola and southern Tunisia, were in the lowest-prevalence decile in 2000 and 2017.

**Fig. 2:**
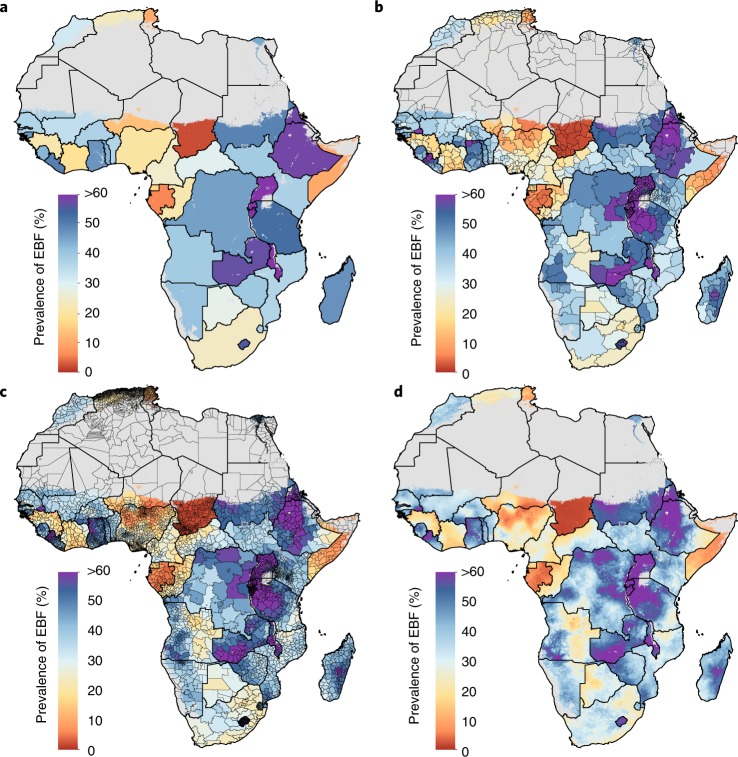
EBF prevalence in 2017 among infants under 6 months at different levels of spatial resolution. **a**–**d**, Prevalence of EBF in 2017 at the national (**a**), first administrative subdivision (**b**), second administrative subdivision (**c**) and 5 km × 5 km grid cell level (**d**). Maps reflect administrative boundaries, land cover, lakes and population; gray-colored grid cells had fewer than ten people per 1 km × 1 km grid cell and were classified as ‘barren or sparsely vegetated’, or were not included in this analysis.

Our detailed spatial estimates display broad within-country differences throughout Africa that would otherwise be masked by national or less granular subnational estimates. ‘Hot spots’ of low EBF prevalence are highlighted at higher resolutions (Fig. 2). Nationally in 2017, Ethiopia (58.2% (50.4–65.8%)), Tanzania (52.6% (46.0–58.9%)), DRC (45.9% (40.0–52.5%)), Kenya (37.6% (26.8–49.5%)) and Namibia (40.9% (31.6–50.2%)) were at or approaching the 2025 prevalence target (see Fig. 1f for a relative uncertainty map). However, some second administrative subdivisions in south-eastern Ethiopia and Tanzania with slower EBF uptake fell short, and will fail to meet targets based on current trajectories (Supplementary Tables 11 and 12), while local-level areas in north-eastern Namibia and south-western DRC and Kenya were found to have lower prevalence (<25%). Within-country disparities in estimated EBF prevalence were both common and widespread: in 2017, at least a twofold difference in estimated EBF prevalence existed across second administrative subdivisions in 53.1% (26 of 49) of African countries; at least a threefold difference occurred in 14.3% (7 of 49) of countries, and a more than sixfold difference was estimated in Niger and Nigeria.

**Fig. 3:**
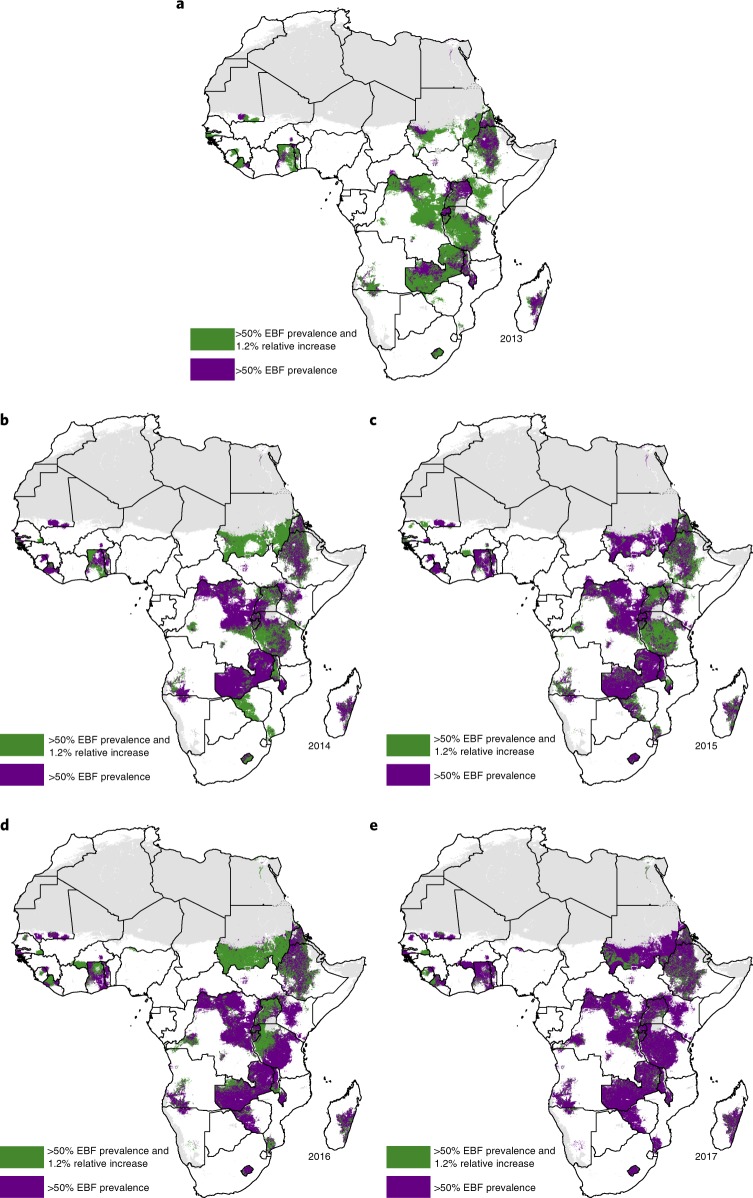
Progress towards WHO GNT 2025 during 2013–2017. **a**–**e**, Results are shown for 2013 (**a**), 2014 (**b**), 2015 (**c**), 2016 (**d**) and 2017 (**e**). Areas in purple highlight places that met WHO GNT by achieving at least 50% EBF prevalence. Areas in green highlight locations that achieved a 1.2% annual relative increase in addition to meeting WHO GNT of at least 50% EBF prevalence. Maps reflect administrative boundaries, land cover, lakes and population; gray-colored grid cells had fewer than ten people per 1 km × 1 km grid cell and were classified as ‘barren or sparsely vegetated’, or were not included in this analysis.

The weighted AROC between 2000 and 2017 (Fig. 1g) and corresponding projected levels of EBF prevalence in 2025 (Fig. 1h) were highly variable across the continent, with declines in EBF prevalence observed in several countries. In some cases—as in Madagascar—these decreases occurred against a background of initially high EBF prevalence in 2000, and a few central areas are nonetheless projected to meet WHO GNT of at least 50% EBF by 2025, assuming that recent trends continue. Although Ethiopia’s north-western areas met WHO GNT by 2017, some of these locations experienced annualized declines and failed to meet the minimum 1.2% relative annual increase recommended for well-performing countries^6^ (Fig. 3). Following current trajectories, fewer than half of African countries (36.7%; 18 of 49) are projected to meet or exceed WHO GNT by 2025 based on national-level estimates (Supplementary Table 12). Success in meeting WHO GNT in 2025 was predicted for all first administrative subdivisions in just eight countries (Burundi, Guinea-Bissau, Lesotho, Malawi, Rwanda, São Tomé and Príncipe, Sierra Leone and Zambia) and for all second administrative subdivisions in just three countries (Guinea-Bissau, Rwanda, and São Tomé and Príncipe) (Fig. 3 and Supplementary Table 12).

**Fig. 4:**
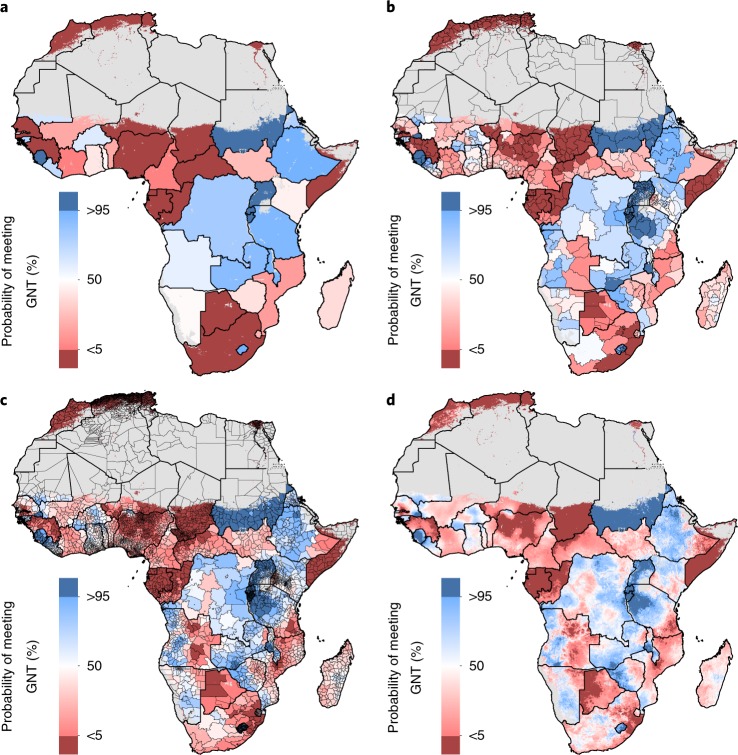
Probability of meeting WHO GNT for EBF by 2025 at different levels of spatial resolution. **a**–**d**, Probability of meeting WHO GNT of at least 50% EBF prevalence by 2025 at the national (**a**), first administrative subdivision (**b**), second administrative subdivision (**c**) and 5 km × 5 km grid cell level (**d**). Maps reflect administrative boundaries, land cover, lakes and population; gray-colored grid cells had fewer than ten people per 1 km × 1 km grid cell and were classified as ‘barren or sparsely vegetated’, or were not included in this analysis.

Many areas will require substantial acceleration of current rates of improvement or reversals in trends to meet WHO GNT, including much of North Africa and western SSA, and parts of all other African regions (Fig. 1i). Given the rates of change we estimated for the 2000–2017 period, a band of areas along the Sahel in western SSA need an estimated 400% or more increase of existing AROC to achieve 50% EBF prevalence by 2025. Most East African Rift and bordering countries are on track to achieve targets. Despite large gains between 2000 and 2017, and high AROC, reaching WHO GNT remains unlikely (<50% probability) by projections for some countries, such as in Mali and Côte d’Ivoire (Fig. 4). At subnational levels, just 6.3% (412 of 6,499) of second administrative subdivisions across Africa have a high probability (>95%) of reaching WHO GNT by 2025, while 43.3% (2,817 of 6,499) were almost certain to not reach the target (<5% probability). Local-level variation of this probability can be broad; within Senegal, Angola, Ethiopia and Tanzania, areas with <5% and areas with >95% probabilities of meeting WHO GNT were estimated. Despite a higher probability (>50%) of national achievement to meet the 50% prevalence target by 2025, this goal was not within reach in Ethiopia’s or Tanzania’s south-eastern areas (<5% probability), indicating vulnerable populations left behind in general progress.

**Extended Data Fig. 1:**
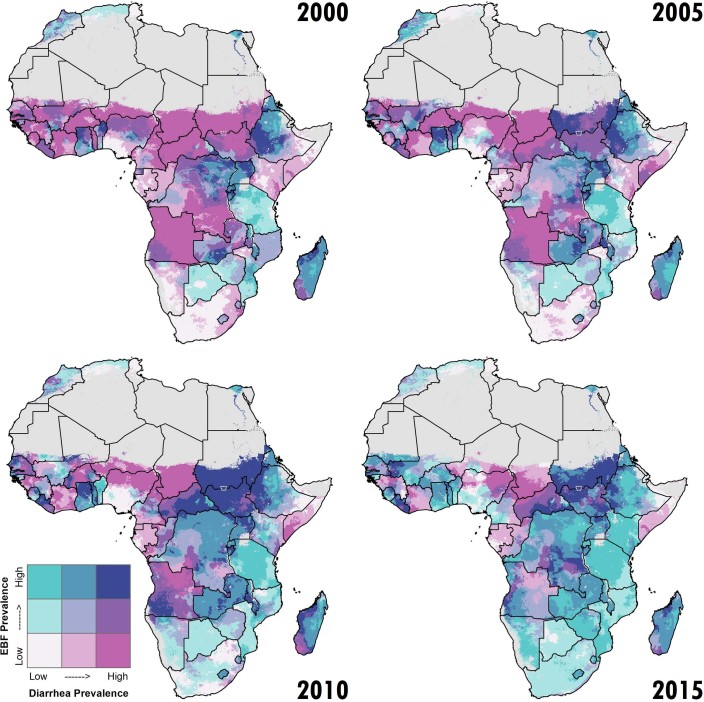
Comparison of diarrhea prevalence in children under 5 years and EBF prevalence by area. Overlapping population-weighted tertiles of diarrhea prevalence (in children under 5 years)^12^ and EBF prevalence (in children 0–5 months) in 2000, 2005, 2010 and 2015. Cut-offs for the tertiles were 20.8 and 36.1% for the EBF prevalence axis, and 3.6 and 5.0% for the diarrhea prevalence axis. Maps reflect administrative boundaries, land cover, lakes and population; gray-colored grid cells had fewer than ten people per 1 km × 1 km grid cell and were classified as ‘barren or sparsely vegetated’, or were not included in this analysis.

**Extended Data Fig. 2:**
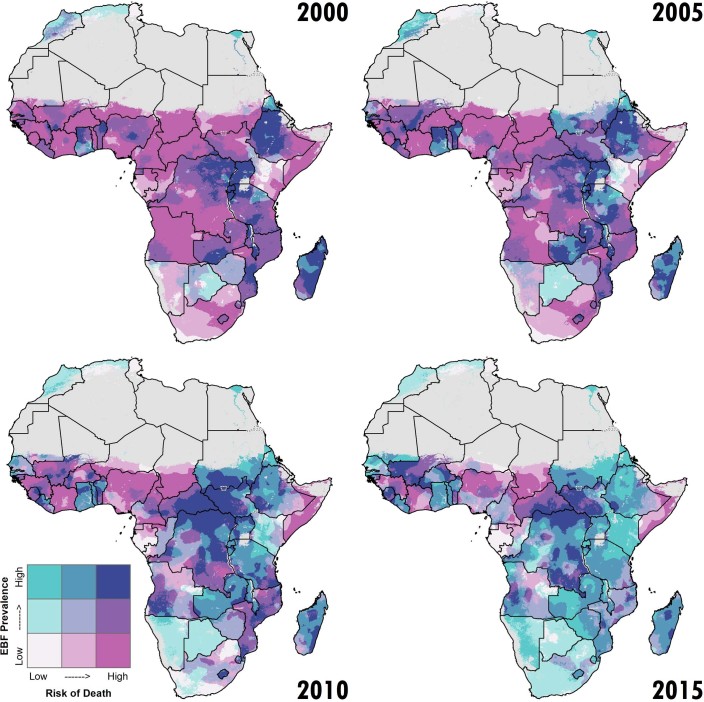
Comparison of risk of death (age 0–11 months) and EBF prevalence by area. Overlapping population-weighted tertiles of mortality risk (in children 0–11 months)^13^ and EBF prevalence (in children 0–5 months) in 2000, 2005, 2010 and 2015. Cut-offs for the tertiles were 20.8 and 36.1% for the EBF prevalence axis, and 4.3 and 6.4% for the risk of death axis. Maps reflect administrative boundaries, land cover, lakes and population; gray-colored grid cells had fewer than ten people per 1 km × 1 km grid cell and were classified as ‘barren or sparsely vegetated’, or were not included in this analysis.

These geospatial analyses add to the landscape of mapping progress towards nutrition targets and, when compared against mapped estimates of conditions associated with infant nutrition, can aid in determining where the most at-risk populations are located. Many child health conditions are inextricably linked to infants’ feeding practices. EBF is associated with reduced incidence of diarrhea and pneumonia, and reduced infant mortality rates^2^. Areas with low EBF prevalence and high diarrheal incidence^12^, such as in communities scattered throughout western and central SSA and Somalia in 2015 (Extended Data Fig. 1), could benefit from increased breastfeeding promotion, education and support. Locations with both high infant mortality^13^ and low EBF prevalence (Extended Data Fig. 2), such as in Somalia, and along or near the western Sahel in Guinea, Côte d’Ivoire, Nigeria, Chad and CAR require urgent attention to improve EBF practices for the greatest benefit to child survival.

Our results underscore substantial improvements in EBF practices across large geographic areas, as well as disparities both between and within countries. Overall, we estimated that only 18 African countries out of 49 (36.7%) are nationally on track to meet WHO GNT by 2025, agreeing with the United Nations Children’s Fund (UNICEF) and WHO conclusions that the majority of nations are not on track to achieve nutrition targets^8,9^. Our projections offer the additional capacity to identify which countries and areas are predicted to fall short of WHO GNT, with estimates disentangled from the tracking of other nutritional targets. Even within countries projected to meet WHO GNT, pockets of slow uptake remain; only three countries are predicted to reach 50% EBF prevalence by 2025 in all second administrative subdivisions, and each region had countries with poor-performing areas (<25% estimated prevalence). By mapping local-level EBF prevalence and trends, we reveal considerable geographic heterogeneity, provide a tool to aid decision-makers in visualizing where populations with the greatest needs may reside, and allow for the aggregation of estimates within meaningful catchment areas or administrative levels. When compared against additional data on interventions employed, this information could aid in identifying program or policy successes and failures.

The varied success in implementing national policies—including the adoption and enforcement of the International Code of Marketing Breast-Milk Substitutes^14^ (the Code), paid maternity leave and breastfeeding-related workplace programs^15^, and the Baby-Friendly Hospital Initiative (BFHI)^16^—may contribute to variation in EBF levels across Africa. In 1973, the publication of *The Baby Killer*, an exposé on the manipulative marketing tactics used by some breast-milk substitute (BMS) companies, raised widespread alarm^17^. Controversial BMS promotion strategies, such as free samples and dressing saleswomen as nurses, may have led to increased usage and dependence on unaffordable formula products lacking the nutritional and immunological benefits of breast-milk, and the exposure of infants to increased risks of pathogens and mortality^17^. In 1981, the Code^14^ was created at the World Health Assembly to encourage breastfeeding and regulation of the safety and promotion of alternatives. However, as of 2018, only 12 African countries have comprehensive legislation on the Code, and 17 of 47 have no legal measures in place to protect consumers from aggressive BMS marketing^18^. Even with provisions enacted into national law, active monitoring systems and implementation may be weak or inadequate, yet evidence suggests that more restrictive policies may be associated with less pervasive promotion of unnecessary BMS usage^19,20^. Additional legislation and more thorough enforcement of existing legislation is needed in some countries.

Maternal protection policies—such as onsite child care, physical areas for breastfeeding or pumping and storing breast-milk, and paid maternity leave policies—offer additional support and autonomy to mothers working outside the home who may otherwise turn to BMS^19,21–23^. The International Labour Organization advocates for legislation requiring 14 weeks of paid maternity leave to support breastfeeding by working mothers^9^. Additionally, a recent analysis across 38 low- and middle-income countries found a significant and positive association between a 1-month increase in legislated maternity leave and a 5.9 percentage-point difference in EBF^15^. A study in Ghana showed that working mothers with shorter maternity leave were less likely to practice EBF^24^. In 2018, however, only ten African countries met the basic provisions of maternity leave^25^.

BFHI is a WHO- and UNICEF-led effort to ensure that all hospital-based and free-standing maternity units are breastfeeding support centers^16^. ‘Baby friendly’-designated facilities do not accept free or low-cost BMS, and implement ten steps for successful breastfeeding—a package of clinical practices and management procedures that have been demonstrated to improve EBF rates^26^. While the majority of African countries have adopted BFHI, only two have reported more than 50% implementation of the ten steps across their facilities^25^. Many have reported 0% implementation or have not assessed facilities in the past 5 years, suggesting that the initiative has become dormant^9,25^. As with any program, BFHI delivery efficiency varies across space and time; thus, local-level monitoring to gauge progress is needed.

Many of EBF’s primary barriers involve cultural perceptions and misinformation, which can be highly variable across communities, contributing to local variation in EBF practices and the need for community-based interventions. Women’s perceptions of insufficient breast-milk, beliefs about infant thirst and need for water, and the cultural and family norms that support the early introduction of food and liquid are a few examples of barriers that vary broadly across communities^21–23,27^. Mothers who perceive their breast-milk to be nutritionally inadequate are more likely to discontinue EBF^22^. In many settings, mothers’ early breast-milk (colostrum) is considered sour and difficult to digest, and is discarded and replaced by prelacteal feeding of water, formula or animal milk, making it difficult to establish breastfeeding^21–23^. The early introduction of water and porridges is common practice across the continent^23,28^, inhibiting EBF practice and exposing infants to disease and nutritional risks from pathogens; plain water is the greatest obstacle in the western and central regions^28^, and women along the Sahel have cited the high heat index as a reason for feeding their infants water^23^. Generational feeding practices are passed on, and mothers can be influenced by community and family members’ attitudes towards breastfeeding^23,28,29^.

Although pervasive, the aforementioned issues can be addressed through lactation management, breastfeeding support, and social and behavior-change communication approaches^27^. Through intensive home-based support or participatory women’s groups, health workers can dispel breastfeeding myths, increase confidence and equip mothers with the necessary skills to address breastfeeding issues, including infant suckling difficulties or pain^22^. A study in Ghana showed that women who received infant feeding recommendations from health workers were more likely to practice EBF^24^. An integrated approach combining promotion, counseling and education on EBF in communities and health facilities has been found to be significantly more effective than counselling as a single intervention^29^. In a systematic review of 46 studies, all forms of extra breastfeeding support—including face-to-face or telephone interactions by professional or lay support staff—led to a decrease in EBF cessation (risk ratio 0.88 (0.85–0.92)) when analyzed together^30^. As of 2018, however, only 18 of the 49 African countries in our analyses offered community-based breastfeeding programs in all of their districts, and 21 report offering individual infant and young child feeding counselling in all of their primary health care facilities^25^; no information on the quality of services or number of women reached by these programs is available^9^. Funding for such interventions is limited, as only 17 African countries currently receive at least US$2 per birth towards breastfeeding programs^25^.

Government buy-in and combined approaches are key to increasing the likelihood of success of community-based programs. The Alive & Thrive Initiative has shown that improving EBF is possible at scale through a combination of advocacy, interpersonal communication, community mobilization and mass media^21,31^. In Ghana and Madagascar, EBF rates significantly improved when training, as well as social and behavior-change activities, were delivered via partnerships between government and non-governmental organizations^27^. Furthermore, a meta-analysis found that combining health system, home and community-based approaches was most effective at improving EBF rates^29^. Impact evaluations of similar community-based projects with health systems integration in Ethiopia, Kenya and Senegal found EBF counselling from both facility-based and community personnel to significantly increase the odds (odds ratio = 2.90; *P* < 0.001) of EBF^32^.

It is difficult to interpret EBF trends in Africa without considering the human immunodeficiency virus (HIV) epidemic and the impact that evolving recommendations may have had on infant feeding practices in high-burden countries. In 1997, WHO was advising HIV-infected mothers to avoid breastfeeding—to prevent mother-to-child transmission—if replacement feeding could be practised safely^33^. However, several studies in the early 2000s reported lower HIV transmission risk among exclusively breastfed infants compared with those who were mixed-fed (that is, breastfed and given solid foods or infant formula)^34–36^. These studies, coupled with subsequent research indicating the efficacy of antiretroviral therapy (ART)^37^, led to new guidance in 2010 in favor of EBF for children of HIV-infected women on ART^38^—a recommendation reiterated in 2016^39^. These changing recommendations may have contributed to low initial EBF rates in 2000 and subsequent improvements through 2017 in some countries, particularly in eastern and southern SSA, where HIV prevalence is high and access to HIV testing, counselling and ART increased during the 2000–2017 period. Continued access to HIV treatment, along with support for breastfeeding in high-burden areas, are urgent priorities to further EBF and optimize maternal and child health^40^.

Widely considered one of the most effective behaviors in preventing child mortality, and a key component of WHO’s Global Action Plan for the Prevention and Control of Pneumonia and Diarrhoea^1^, EBF can save and improve the quality of lives^3^. While facilities may be collecting data on breastfeeding interventions and EBF rates for monitoring purposes in some locations, improved data collection efforts are needed across many African locales, and the estimates here are supplemental to those efforts. The EBF estimates presented here, at various spatial resolutions, can assist public health practitioners and policymakers in visualizing and identifying disparities across and within countries—informing decisions on where existing interventions and policies may need to be bolstered, or new strategies considered, to ensure that all infants have the opportunity to survive and thrive.

## Methods

### Overview

Our analyses provide annual estimates of EBF prevalence among infants under 6 months of age during the period of 2000–2017 across Africa at the national, first and second administrative (for example, state and district level, respectively), and 5 km × 5 km grid cell levels. EBF prevalence is defined as the proportion of children who receive only breast-milk, oral rehydration salts or other medicines or vitamins, without receiving additional food or drink (including water) between birth and 6 months of age. Our primary goal was to provide prevalence predictions at a high spatial resolution across the African continent with the best out-of-sample predictive performance. The methodology used here is similar to that used for previous analyses of diarrhea incidence^12^, under 5 years mortality^13^, child growth failure^41^, educational attainment^42^ and HIV^43^ in Africa. We first mapped our estimates on a 5 km × 5 km grid to remain consistent with these previous analyses, align with the resolutions available for pre-existing covariates incorporated in these analyses, and maintain flexibility in aggregating these estimates to other levels of interest (for example, first and second administrative subdivisions). Our analyses of 49 countries include mainland Africa and the islands of Madagascar, Comoros, and São Tomé and Príncipe. We do not provide estimates for Libya, Djibouti or island nations where survey data were not available (Mauritius, Seychelles and Cape Verde). This study follows the Guidelines for Accurate and Transparent Health Estimates Reporting (http://gather-statement.org; Supplementary Table 1).

### Data extraction and processing

**Extended Data Fig. 3:**
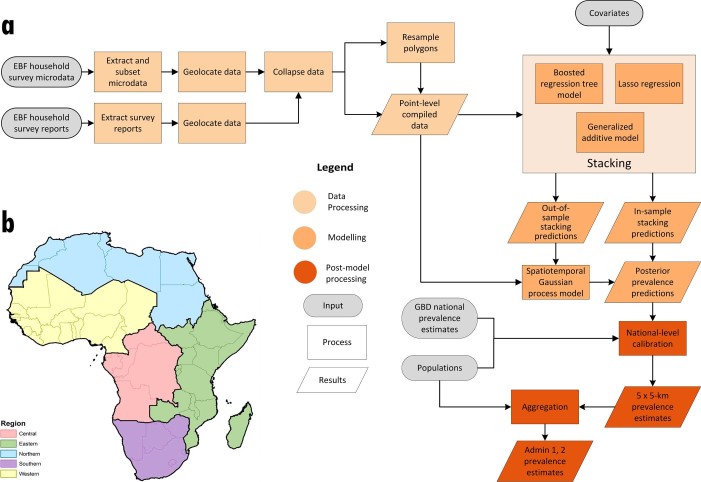
Analytic process overview and map of modeling regions. **a**, The process used to produce EBF prevalence estimates in Africa involved three main parts. In the data-processing steps (peach), data were identified, extracted and prepared for use in the model. In the modeling phase (orange), we used these data and covariates in a stacked generalization ensemble model and spatiotemporal Gaussian process model. In post-processing (red), we calibrated the prevalence estimates to match GBD 2017^5^ estimates and aggregated the estimates to the first- and second-level administrative subdivisions in each country. **b**, Modeling regions were defined as the five GBD regions for Africa: central (central SSA), east (eastern SSA), north (North Africa and the Middle East), south (southern SSA) and west (western SSA). As this study was limited to mainland Africa and African island nations (except Mauritius, Seychelles, Cape Verde Islands, Libya and Djibouti, where relevant data were not available or did not meet our inclusion and exclusion criteria), Middle East countries were excluded (Afghanistan, Bahrain, Iran, Iraq, Jordan, Kuwait, Lebanon, Oman, Palestine, Qatar, Saudi Arabia, Syria, Turkey, United Arab Emirates and Yemen).

Extended Data Fig. 3a describes the detailed steps performed during the data extraction and data processing workflow. We extracted data from the Demographic and Health Surveys (DHS) program, UNICEF's Multiple Indicator Cluster Surveys (MICS), and country-specific and other multinational surveys conducted in the years 1998–2017 for African countries. Though we model estimates for the years 2000 to 2017, we assigned data from 14 surveys in the years 1998–1999 to the year 2000 to address data scaricity in earlier years and to help establish a baseline. We searched the Global Health Data Exchange (GHDx: http://ghdx.healthdata.org/) for all surveys in African countries tagged as containing EBF indicators of interest; designed and tested a codebook, or survey data extraction framework, for breastfeeding variables present in the household surveys; extracted and geo-matched (either to geospatial coordinates or administrative subdivisions) all surveys available for Africa; and refreshed our query of the GHDx for surveys performed in African countries.

#### Data inclusion and exclusion criteria

As our goal was to estimate the prevalence of EBF among infants under 6 months of age, we only included data regarding the feeding of children less than 6 months old at the time of survey (0–5 months in survey data). Specifically, our inclusion criteria for survey microdata (that is, surveys with individual-level responses) were the following: (1) the survey must have been conducted in an African country between 1998 and 2017; (2) survey responses must be available at the individual level; (3) the survey must contain subnational geographic identifiers, which could include either subnational areal units (typically administrative subdivisions) or Global Positioning System (GPS) coordinates, and data referenced to subnational units must also contain survey weights for each observation; and (4) the survey must contain questions about the age of the child, whether the child is still being breastfed and whether the child has consumed other food or liquid items. Typically, consumption during the past 24 h was recorded. In eight out of 181 surveys with microdata, the question about food or liquid items did not specify a particular recall period. After performing sensitivity analysis, we decided to keep those eight surveys in our model. In cases where survey microdata were not available, we searched for survey report estimates. Our inclusion criteria for these survey reports were the following: (1) the survey must have been conducted in an African country between 1998 and 2017; (2) the survey must contain subnational identifiers, which could include subnational areal units (typically administrative subdivisions); and (3) the survey must contain the prevalence of EBF, with a sample size or the lower and upper bounds for the 95% confidence interval.

Very few surveys directly asked about EBF practice; as such, we derived breastfeeding status from questions asking about the consumption of breast-milk and other foods, liquids and medicines consumed in a set period before the survey, typically within the 24-h period before survey completion. We excluded surveys that only asked mothers and caregivers whether infants had been exclusively breastfed (for example, ‘did you exclusively breastfeed?’) without ascertaining further information. This exclusion criterion was established after finding, by comparing the responses in surveys containing both types of questions, that many mothers and caregivers stated that infants had exclusively breastfed but also answered that they had received food or water in the 24-h recall questions. This may have been due to the respondent misunderstanding the meaning of ‘exclusive breastfeeding’, or the question may have been misinterpreted with translation. Instead, we classified children as exclusively breastfed if survey responses indicated that they received only breast-milk and medicines (oral rehydration salts, vitamins or other medicines) without other foods or liquids during the 24-h period before the survey.

To identify potential survey biases, we reviewed national-level survey estimates for each country and compared them with national-level estimates from the DHS program, the 2017 Global Burden of Disease (GBD) study^5^ and the geospatial model. In cases where a survey’s estimates appeared implausible compared with other existing survey-based data sources, we inspected differences in definitions, data collection or other methodological explanations.

#### Identified data sources

**Extended Data Fig. 4:**
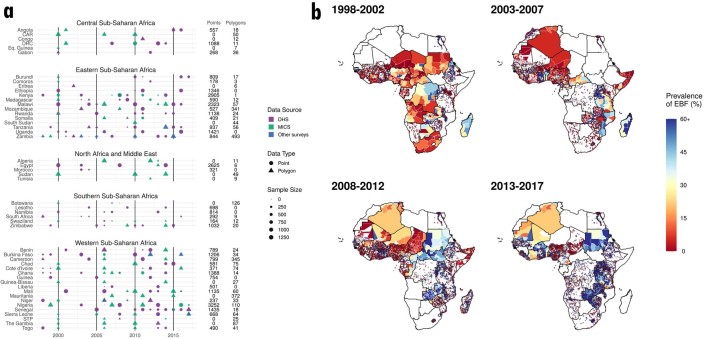
Data availability for EBF among infants under 6 months by type and country, 1998–2017. **a**, EBF data used in this study, by region and country. Color indicates the data source: DHS; MICS; or other survey type. Shape type indicates whether a data source has point (GPS) or polygon (for example, aggregated to an administrative level) location information. Size indicates the relative effective sample size for each source. A full list of data sources, with additional details about data type (such as survey microdata and survey reports) and geographical details, is provided in Supplementary Tables 2 and 3. **b**, Maps of EBF data coverage displayed at 5-year intervals. Maps show the spatial resolution of the underlying data in our models, and the color indicates the EBF prevalence as estimated from the data sources. Countries in white have no available survey data in the given time range.

As a result, we identified and used 188 household surveys that had complete records of questions relating to infant feeding and geographical information; 102 were from the DHS series, 79 were from the MICS series and seven were from other sources. Extended Data Fig. 4 shows the spatial and temporal extent of data availability by country, and Supplementary Tables 2 and 3 provide information on the names, citations and geographic detail of surveys of the underlying data sources of our models.

Supplementary Table 4 provides a list of surveys that were excluded from both the geostatistical model and GBD 2017 estimates^5^. Supplementary Table 5 provides a list of surveys that were included in the geostatistical model but excluded from the GBD estimates (in cases where surveys were non-nationally representative but could provide spatial information for the geostatistical model). Supplementary Table 6 provides a list of surveys that were included in GBD estimates but excluded from the geostatistical model.

#### Data processing

After data identification and extraction, we aggregated the individual-level responses from survey microdata to calculate EBF prevalence and the effective sample size at the finest possible spatial resolution available, incorporating individual-level sample weights and using the Kish approximation^44^ for the effective sample size. Each individual child record was associated with a cluster, a group of neighboring households or a ‘village’ that acts as a primary sampling unit (a census enumeration area). For surveys where a latitude and longitude pair representing the location of each survey cluster were available (‘point data’), data were aggregated to these specific coordinates. Geographic coordinates or place names for each cluster were included in 101 surveys (33,341 clusters).

In the case of survey microdata where geographical coordinates were not available and in the case of survey reports, we assigned data to the smallest available administrative unit in the survey (‘polygon data’)^45^. We ‘resampled’ data matched to polygons to generate pseudo-point data based on the underlying population distribution within the polygon. The methods for resampling were consistent with those previously used in geospatial modeling of under 5 years mortality^13^. Specifically, for each polygon-level observation, we randomly sampled 10,000 locations among grid cells in the given polygon with probability proportional to grid cell population. A grid cell was assigned to a polygon if its centroid fell within the geographic boundary. We performed *k*-means clustering (with *k* set to 1 per 40 grid cells) on the sampled points to generate a reduced set of locations to be used in modeling based on the *k*-means cluster centroids. Weights were assigned to each pseudo-point proportional to the number of sampled points contained in each of the *k*-means clusters (that is, the number of sampled points divided by 10,000). Each pseudo-point generated by this process was assigned the EBF prevalence and sample size observed for the polygon as a whole, and the weights associated with each pseudo-point were applied during all stages of model fitting.

After performing the data processing described above, our final dataset consisted of 60,083 clusters (33,341 of which were GPS-located data points and 26,742 of which were polygon data) from 188 surveys (181 surveys with microdata and seven survey reports) representing 153,465 children across 49 African countries.

### Statistical analysis

#### Covariates

In these analyses, we included the following socioeconomic, environmental and health-related covariates to improve the predictions of EBF: urbanicity, night-time lights, travel time to the nearest settlement with >50,000 inhabitants, total population, human development index (HDI), educational attainment in women of reproductive age (15–49 years old), nutritional yield for vitamin A, and HIV prevalence. These covariates were selected because they are factors or proxies for factors that previous literature has identified to be associated (not necessarily causally) with EBF prevalence.

The first four covariates were included as measures or proxies for connectedness and urbanicity, as EBF is typically found to be different in urban areas compared with rural locations. HDI—a composite indicator of key aspects of development (namely, education, economy and health)—was chosen based on previous studies relating country development to EBF. Educational attainment in women of reproductive age (15–49 years old) was included because previous studies highlight education as a maternal factor influencing the decision to initiate and continue EBF. Nutritional yield for vitamin A was chosen as a proxy of maternal nutrition while breastfeeding. HIV was included given the known risks of mother-to-child transmission of HIV and consequent potential avoidance of breastfeeding in hyper-endemic settings over the study period. These covariates underwent spatial and temporal processing in preparation for their inclusion in analysis. See Supplementary Table 7 for references to the covariate data used in the models, as well as references supporting our rationale for using these covariates.

Spatial processing involved resampling the input covariate raster to align the spatial resolution of the covariate to the 5 km × 5 km resolution used in modeling. For covariates that were originally at a finer resolution, we resampled the raster by taking the neighborhood average (that is, for the covariates ‘travel time to the nearest settlement of >50,000 inhabitants’ and ‘night-time lights’) or using the nearest neighbor (that is, for the covariate ‘urbanicity’) or sum (that is, for the covariate ‘total population’) of the finer covariate raster to produce one at a 5 km × 5 km resolution. Educational attainment in women of reproductive age and HIV covariates were produced at a 5 km × 5 km resolution in our previous studies, and thus did not require additional spatial processing. For covariates that were originally at lower resolutions (that is, the covariates ‘HDI’ and ‘nutritional yield for vitamin A’), we resampled the raster using bilinear interpolation, with the effect of smoothing some of the hard pixel boundaries in the raw data to make for a 5-km × 5-km-resolution raster.

Temporal processing was required in instances where the original temporal resolution of the covariate was anything other than annual. To resolve from a coarser time period to an annual time period, we filled the intervening years with the value from the nearest neighboring year (that is, for the covariate ‘urbanicity’) or used an exponential growth rate model (that is, for the covariate ‘total population’). Night-time lights, educational attainment and HIV prevalence were available at a 1-year temporal resolution and did not require interpolation. As the travel time to the nearest settlement of >50,000 inhabitants and nutritional yield for vitamin A covariates were available only for a single representative year (2015 and 2005, respectively), these covariates were set to be unchanged over time. After interpolation, the covariates of night-time lights, HDI and urbanicity were still missing information for the most recent years of the 2000–2017 period, and in these instances we filled out the end of the time series carrying forward the most recent year without modification.

**Extended Data Fig. 5:**
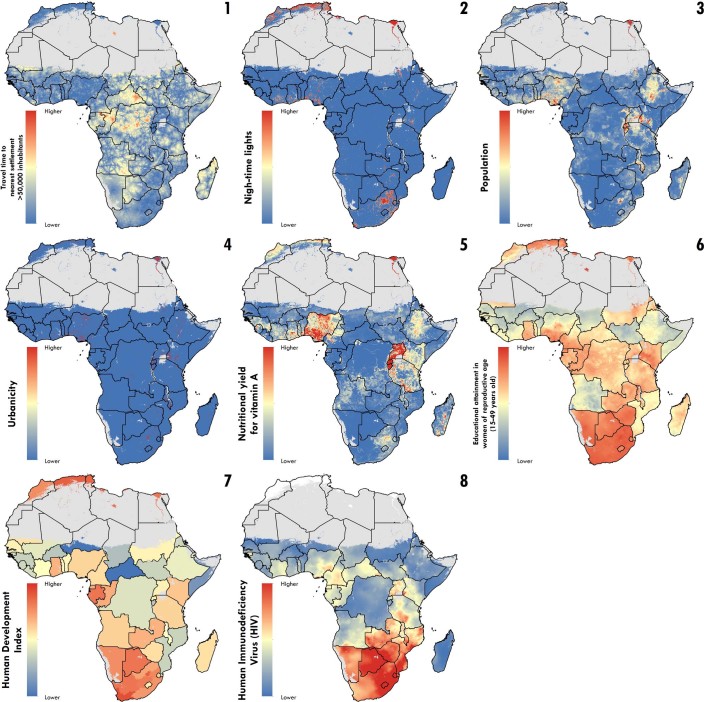
Map of covariates. Covariate raster layers of possible socioeconomic, environmental and health-related covariates used as inputs for the stacking modeling process. Time-varying covariates are presented for the year 2017. For additional detail on the year of production of non-time-varying covariates, see the individual covariate citation in Supplementary Table 7. Maps reflect administrative boundaries, land cover, lakes and population; gray-colored grid cells had fewer than ten people per 1 km × 1 km grid cell and were classified as ‘barren or sparsely vegetated’, or were not included in this analysis.

We list detailed information on the temporal resolution and source(s) for each of the eight included covariates in Supplementary Table 7. In addition, the calendar year was used as a covariate in our model. See Extended Data Fig. 5 for maps of spatial covariate raster layers for 2017.

#### Spatial covariate stacking

Our primary goal was to provide prevalence predictions across the African continent at a high resolution, and we used methods designed to provide the best out-of-sample predictive performance at the cost of inferential understanding. An ensemble covariate modeling method was implemented to both select covariates and capture possible nonlinear effects and complex interactions between them^46^. We fit separate models for five African regions based on the geographical regions defined for the GBD^47^ (central, eastern, northern, southern or western, as seen in Extended Data Fig. 3b). For each region, three submodels were fitted to our dataset, using all of our covariate data as explanatory predictors: generalized additive models, boosted regression trees and lasso regression. We selected these three submodels based on the ease of implementation through existing software packages, the fundamental differences in their approaches and a proven track record in predictive accuracy^46^. Submodels were fit in R using the mgcv, xgboost, glmnet and caret packages.

**Extended Data Fig. 6:**
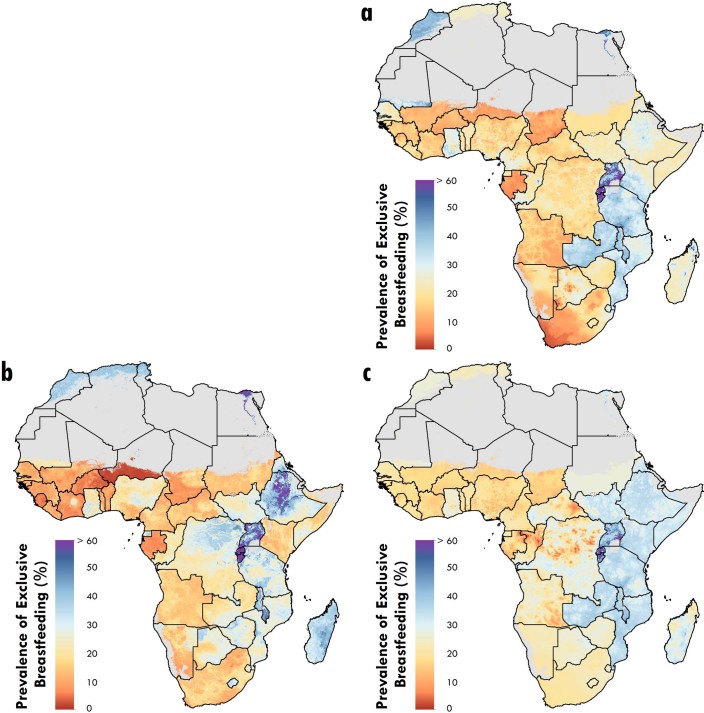
Maps of in-sample predictions from the ensemble covariate modeling process. **a**–**c**, Each map represents the in-sample predicted prevalence of EBF generated from the three submodels: (**a**) a generalized additive model; (**b**) a boosted regression trees model; and (**c**) lasso regression, for 2000. Maps reflect administrative boundaries, land cover, lakes and population; gray-colored grid cells had fewer than ten people per 1 km × 1 km grid cell and were classified as ‘barren or sparsely vegetated’, or were not included in this analysis.

Each submodel was fit using fivefold cross-validation to avoid overfitting, and hyper-parameter fitting was performed to maximize the predictive power. For each submodel, we produced two sets of predictions: out of sample and in sample. Out-of-sample predictions for each model were generated by compiling the predictions from the five holdouts from each cross-validation fold, and in-sample predictions were generated by refitting the submodels using all available data. The out-of-sample submodel predictions were used as explanatory covariates when fitting the geostatistical model described below, and the in-sample predictions were used when generating predictions from the geostatistical model, to maximize data use. In both cases, the logit transformation of the predictions was used to put these predictions on the same scale as the linear predictor in the geostatistical model. Maps of in-sample predictions from each stacker are presented in Extended Data Fig. 6. A recent study has shown that this ensemble approach can improve predictive validity by up to 25% over an individual model^46^.

#### Geostatistical model

As a second step, we fit the geostatistical model below separately for the five African regions. For each region, we write the hierarchy that defines our Bayesian model as follows:

$$\begin{array}{l}{\rm{EBF}}_i\left| {p_i,N_i\sim {\mathrm{binomial}}({p_i,N_i})} \right.\\ {\mathrm{logit}}\left({p_i} \right) = {\beta }_0 + {\mathbf{X}}_i\,{\boldsymbol{\beta}}+ \gamma _{ci} + {\it{\epsilon }}_{{\rm{GP}}i} + {\it{\epsilon }}_i\\ {\sum} {\boldsymbol{\beta}= 1} \\ \gamma _{ci}\sim {\rm{normal}}( {0,\,{\it{\sigma}} _{{\rm{country}}}^2})\\ {\it{\epsilon }}_i\sim {\rm{normal}}({0,\,{\it{\sigma}} _{{\rm{nug}}}^2})\\ {\boldsymbol{\epsilon }}_{{\rm{GP}}}\left| {{\Sigma}_{{\rm{space}}},\,{\Sigma}_{{\rm{time}}}\sim {\mathrm{GP}}({0,\,{\Sigma }_{{\rm{space}}} \otimes {\Sigma }_{{\rm{time}}}})} \right.\end{array}$$

We modeled the number of children who were categorized as ‘exclusively breastfed’ (EBF_*i*_) among a sample size (*N*_*i*_) at space–time location (*i*) as a binomial random variable. The logit-transformed prevalence of EBF (*p*_*i*_) was specified as a linear combination of a regional intercept (*β*_0_), the logit-transformed predictions from the three submodels (***X***_*i*_), country-level random effects (*γ*_*ci*_), a correlated spatiotemporal error term (${\it{\epsilon }}_{{\rm{GP}}i}$) and an independent and identically distributed nugget (uncorrelated error term) effect $\left( {{\it{\epsilon }}_i} \right)$. Weighting coefficients (***β***) were constrained to sum to 1 (ref. ^46^). The spatial covariance (*Σ*_space_) was modeled using an isotropic and stationary Matérn function^48^. The temporal covariance (*Σ*_time_) was an annual first-order autoregressive function.

The intercept captures the overall mean level of EBF prevalence, while the covariate effects capture the spatial and temporal variation in EBF prevalence that can be described as a function of spatial and temporal variation in the included covariates. The country random effects capture additional variation between countries. Spatially and temporally correlated random effects capture additional variation by location (within and between countries) and time. Finally, the uncorrelated error term (or nugget effect) captures any additional, non-structured variation by location and time.

The Matérn covariance function is associated with two hyper-parameters, *κ* and *τ* (*ν* is fixed at 1), while a temporal first-order autoregressive (AR1) covariance function is associated with one hyper-parameter, *ρ*. The following hyper-priors were set for each of these parameters:

$$\begin{array}{l}{\theta}_1 = \log \left[\tau \right]\sim {\mathrm{normal}}({\mu _{\theta _1},\,{\it{\sigma}} _{\theta _1}^2})\\ {\theta }_2 = \log \left[\kappa \right]\sim {\mathrm{normal}}({\mu _{\theta _2},\,{\it{\sigma}} _{\theta _2}^2})\\ {\mathrm{log}}\left[{\left({1 + \rho } \right)/\left({1 - \rho } \right)} \right]\sim {\mathrm{normal}}({4,1.2^2})\end{array}$$

The prior for the temporal correlation parameter, *ρ*, corresponds to a mean of 0.96 and a distribution that is wide enough to include approximately 0.2 to 1.0 within three standard deviations of the mean. This relatively informative prior was chosen because temporal correlation was expected to be high. $\mu _{\theta _1}$, $\sigma _{\theta _1}$, $\mu _{\theta _2}$ and $\sigma _{\theta _2}$ were automatically determined by integrated nested Laplace approximation (INLA). Priors for fixed effects and hyper-priors for other random effects were set as:

$$\begin{array}{l}\beta _0 \sim {\mathrm{normal}}\left( {0,\,3^2} \right)\\ 1/{\it{\sigma}} _{{\rm{country}}}^2 \sim {\mathrm{gamma}}\left( {{\mathrm{rate}} = 1,\,{\mathrm{shape}} = 0.00005} \right)\\ 1/{\it{\sigma}} _{{\rm{nug}}}^2 \sim {\mathrm{gamma}}\left( {{\mathrm{rate}} = 1,\,{\mathrm{shape}} = 0.00005} \right)\end{array}$$

This model was fit in R-INLA^49^ using the stochastic partial differential equations^50^ approach to approximate the continuous spatiotemporal Gaussian random fields $\left( {{\it{\epsilon }}_{{\rm{GP}}i}} \right)$. We constructed a finite-elements mesh for the stochastic partial differential equations approximation to the Gaussian process regression using a simplified polygon boundary (as seen in Supplementary Fig. 12 of our previous publication of geospatial estimates of child growth failure^41^). We set the inner mesh triangle maximum edge length (the mesh size for areas over land) to be 0.25 decimal degrees, and the buffer maximum edge length (the mesh size for areas over the ocean) to be 5.0 decimal degrees. Fitted model parameters are listed in Supplementary Table 8.

After fitting each model based on regional classification, we generated 1,000 draws of all model parameters from the approximated joint posterior distribution using the inla.posterior.sample() function in R-INLA. For each draw, *s*, of the model parameters, we constructed a draw of $p_i^{(s)}$ as:

$$p_i^{(s)} = {\rm{logit}}^{ - 1}\left({{{\beta}}_0^{(s)} + {\mathbf{X}}_i\,{\boldsymbol{\beta}}^{(s)} + {\gamma }_{ci}^{(s)} + {\epsilon }_{{\rm{GP}}i}^{(s)} + {\epsilon }_i^{(s)}} \right)$$

Additional processing of the output from inla.posterior.sample() is required for the correlated spatiotemporal error term $\left( {{\it{\epsilon }}_{{\rm{GP}}i}^{(s)}} \right)$ and the nugget effect $\left( {{\it{\epsilon }}_i^{(s)}} \right)$ before constructing $p_i^{(s)}$ according to the equation above. Specifically, for ${\it{\epsilon }}_{{\rm{GP}}i}^{(s)}$, draws are generated initially only at the vertices of the finite element mesh, so we project from this mesh to each location *i* desired for prediction (that is, the centroid of each grid cell on a 5 km × 5 km grid, as well as years from 2000–2017). For the nugget effect, we generate ${\it{\epsilon }}_i^{(s)}$ for each *i* by sampling from normal $\left( {0,{\it{\sigma}} _{\mathrm{nug}}^{2\quad (s)}} \right)$. At the end of this process, we have 1,000 draws of *p*_*i*_ for each grid cell and year.

#### Model validation

##### Validation strategy

We used fivefold cross-validation to assess the performance of the modeling framework described above. To do so, we first split all survey data into five groups by randomly sorting a list of unique identifiers for each survey, calculating the cumulative effective sample size represented by the surveys in this list, and then dividing the list into five parts at the point where this cumulative sample size was closest to 20, 40, 60 and 80% of the total. This resulted in five groups that were approximately equal in terms of the total effective sample size, and which contained entire surveys (that is, all of the data points derived from each survey were contained exclusively within only one fold). We then fit the model described above five times, excluding each of the five groups of data in turn.

After fitting the model five times, the data withheld from each model were matched with predictions from that model, and then these data–prediction pairs were compiled across all five models, resulting in a complete dataset of out-of-sample predictions corresponding to all survey data included in the analysis. EBF prevalence estimates based on single survey clusters are generally quite noisy due to very small sample sizes, and were consequently insufficient as a ‘gold standard’ for evaluating the model predictions^13^. To address this issue, we aggregated both the observed data and the corresponding out-of-sample predictions within countries and within first- and second-level administrative subdivisions, by calculating a weighted mean of each using the effective sample sizes as the weights. Then, across all data–estimate pairs, we calculated two summary measures: the mean error (a measure of bias) and the root-mean-square error (RMSE; a measure of total variance). In addition, for each data–estimate pair, we constructed 95% prediction intervals from the 2.5th and 97.5th percentiles of 1,000 draws from a binomial distribution corresponding to each of the 1,000 posterior draws of EBF prevalence, with *p* equal to EBF prevalence in a given posterior draw and *N* equal to the effective sample size for the data point type. We then calculated coverage as the percentage of data–estimate pairs where the data point was contained within this 95% prediction interval.

##### Sensitivity analyses

To assess the utility of the stacking ensemble, we ran five fivefold cross-validation holdout experiments, using different combinations of covariates and random effects. The following five models were compared:

1. raw covariates:
  $${\mathrm{logit}}\left({p_i} \right) = {\beta }_0 + {\mathbf{X}}_i\, {\boldsymbol{\beta}} _{{\rm{raw}}} + \gamma _{ci} + \epsilon _i$$
2. stacking predictions as covariates:
  $${\mathrm{logit}}\left({p_i} \right) = {\beta }_0 + {\mathbf{X}}_i\,{\boldsymbol{\beta}}_{{\rm{stack}}} + \gamma _{ci} + \epsilon _i$$
3. a Gaussian process:
  $${\mathrm{logit}}\left({p_i} \right) = \beta_0 + \gamma _{ci} + \epsilon _{{\mathrm{GP}}_i} + \epsilon _i$$
4. raw covariates + a Gaussian process:
  $${\mathrm{logit}}\left({p_i} \right) = \beta_0 + {\mathbf{X}}_i\,{\boldsymbol{\beta}}_{{\rm{raw}}} + \gamma _{ci} + \epsilon _{{\mathrm{GP}}_i} + \epsilon _i$$
5. stacking covariates + a Gaussian process (final model):
  $${\mathrm{logit}}\left({p_{\mathrm{i}}} \right) = \beta _0 + {\mathbf{X}}_i\,{\boldsymbol{\beta}}_{\rm{stack}} + \gamma_{ci} + \epsilon _{{\mathrm{GP}}_i} + \epsilon _i$$

**Extended Data Fig. 7:**
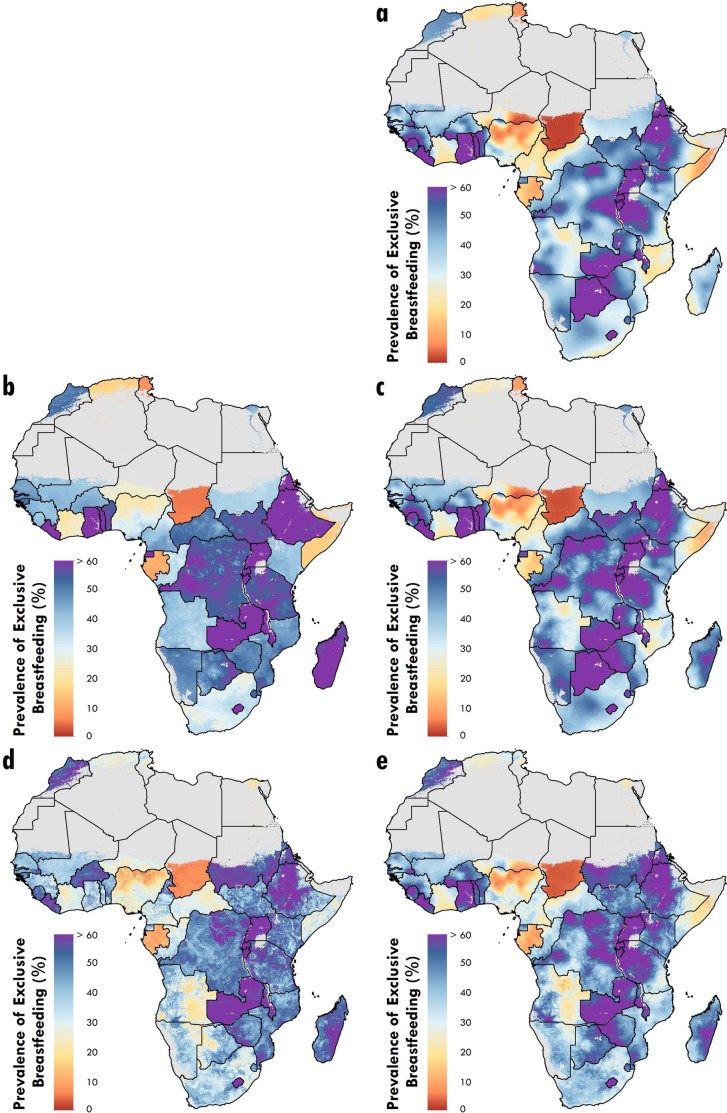
Predictions comparison from the covariate sensitivity analysis. **a**–**e**, EBF prevalence among infants under 6 months in 2017 at the 5 km × 5 km grid cell level, based on models with no covariates and including: a Gaussian process (**a**); raw covariates with no Gaussian process (**b**); raw covariates with a Gaussian process (**c**); stacked covariates with no Gaussian process (**d**) and stacked covariates with a Gaussian process (e; the final model). Estimates are shown without calibration to GBD 2017^5^, to better highlight the differences between the models (that would have been masked after calibration). Maps reflect administrative boundaries, land cover, lakes and population; gray-colored grid cells had fewer than ten people per 1 km × 1 km grid cell and were classified as ‘barren or sparsely vegetated’, or were not included in this analysis.

Supplementary Table 9 compares the results of this cross-validation exercise in terms of the performance of these five different modeling strategies, and Extended Data Fig. 7 provides a comparison of the estimates derived from these different models. At all three levels of aggregation, and both in and out of sample, mean error (bias) is relatively low, ranging from −0.49 to 0.42 percentage points. Out-of-sample RMSE is relatively similar for all five models, while in sample, model 1 (raw covariates only) has noticeably worse RMSE compared with the other models. Overall, model 5 (a two-stage model including stacked regression for the covariates) has the lowest out-of-sample RMSE value across three levels of aggregation. The coverage of the 95% prediction intervals showed that the models with a Gaussian process (models 3, 4 and 5) outperformed those without (models 1 and 2). For all models with a Gaussian process, coverage of the prediction intervals was close to 98% in sample, and between 88 and 92% out of sample. From the results of these sensitivity analyses, we chose model 5: a two-stage model including stacked regression for the covariates.

**Extended Data Fig. 8:**
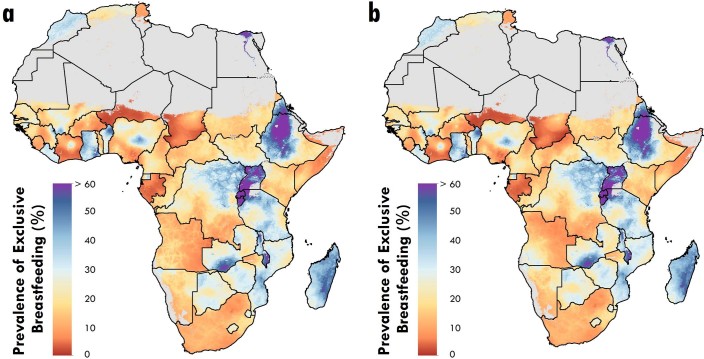
Predictions comparison from the recall period sensitivity analysis. **a**,**b**, EBF prevalence among infants under 6 months in 2000 at the 5 km × 5 km grid cell level, based on models containing only surveys that specify a 24-h recall period (**a**) and containing all available surveys (**b**). Estimates are shown after calibration to GBD 2017^5^, to better highlight the differences between the final maps when the models include all surveys or only surveys that specify a 24-h recall period. Maps reflect administrative boundaries, land cover, lakes and population; gray-colored grid cells had fewer than ten people per 1 km × 1 km grid cell and were classified as ‘barren or sparsely vegetated’, or were not included in this analysis.

Additionally, to assess the impact of including surveys that do not explicitly state a 24-h recall period in questions asking about food and liquid given to a child, we considered models with and without the data included from those surveys. Supplementary Table 9 also compares the cross-validation performance of the two models: one containing only surveys that specify a 24-h recall period; and one containing all available surveys. Both in-sample and out-of-sample metrics are reasonably comparable across these two models. Since the model that includes all surveys does not produce additional bias or underestimate the degree of uncertainty (there were only eight out of 188 surveys that did not specify 24 h as a recall period), we chose to keep all surveys (Extended Data Fig. 8).

#### Post-estimation

**Extended Data Fig. 9:**
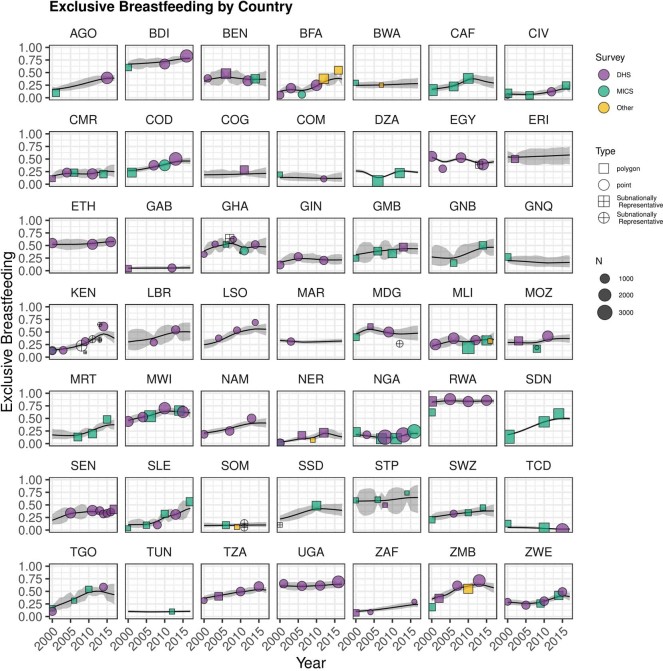
National time series plots and aggregated input data. National time series plots of the post-GBD calibration final estimates by country during 2000–2017. Uncertainty ranges are presented in gray, and aggregated input data are classified by survey series (purple, country specific; green, DHS; yellow, MICS), data type (square, polygon; circle, point) and whether the survey is nationally or subnationally representative. A list of subnationlly representative surveys in given in Supplementary Table 10.

To take advantage of the extensive data gathering and analysis of GBD 2017^5^, which in some cases included data sources outside of the scope of our geospatial modeling framework, we performed post-hoc calibration of our estimates to the GBD estimates^5^ (please refer to Supplementary Tables 2, 3 and 6 for the data sources used). First, each grid cell in our 5 km × 5 km grid was assigned to a GBD geography based on the location of the grid cell centroid. Then, for each country and year, we defined a raking factor that was the ratio of the GBD estimate for this geography and year to the population-weighted posterior mean EBF prevalence across all grid cells within this geography and year. Finally, this raking factor was used to scale each draw of EBF prevalence for each grid cell within the GBD geography and year. The corresponding mean raking factor across all countries was 0.96 (interquartile range: 0.82–1.08), indicating close agreement with GBD estimates. National time series plots of the post-GBD calibration final estimates (including uncertainty ranges) are presented along with the aggregated input data (classified by survey series, data type and sample size) in Extended Data Fig. 9.

After calibration to GBD 2017^5^, grid cell level estimates were aggregated to the second administrative subdivision, first administrative subdivision and national levels using population-weighted averages at the draw level. This was carried out for each of the 1,000 posterior draws (after calibration to GBD 2017^5^, as described above), and then point estimates and uncertainty intervals were derived from the mean, 2.5th percentile and 97.5th percentile of these draws, respectively. In cases where an administrative subdivision did not contain the centroid of any grid cell, the nearest grid cell to it was assigned as its proxy prevalence.

Since the publication of GBD 2017^5^, recently released survey microdata (Senegal 2017, Sierra Leone 2017 and South Africa 2016) and additional survey reports (Algeria 2006, Burkina Faso 2012, Burkina Faso 2016, Mali 2016, Niger 2009, São Tomé and Príncipe 2006, and Somalia 2009) were incorporated to update GBD 2017 estimates using GBD 2017 methods^5^. These updated GBD estimates were used for calibrating our estimates. For additional information on the names, citations and geographic details of these surveys, see Supplementary Tables 2 and 3 (records are marked with a single asterisk).

Although our models can predict for all locations covered by available raster covariates, we applied a mask on barren areas based on Moderate Resolution Imaging Spectroradiometer satellite data^51^. All maps in our figures reflect administrative boundaries, land cover, lakes and population. Gray-colored grid cells represent areas with fewer than ten people per 1 km × 1 km grid cell, and were classified as ‘barren or sparsely vegetated’, or were not included in this analysis^51–55^. This step was intended to be useful to policy planners and data specialists.

#### Projections

We compared our estimated rates of improvement in EBF prevalence over the past 18 years with the improvements needed between 2017 and 2025 to meet WHO GNT (50% EBF prevalence)^6^ by performing a simple projection calculation. First, we calculated log-additive AROC at each grid cell (*i*) by logit-transforming our 18 years of posterior mean prevalence, ${\it{prev}}_{i,{\rm{year}}}^l$ and calculating the AROC between each pair of adjacent years starting with 2001:

$${\rm{\it{AROC}}}_{i,{\rm{year}}}^{l} = {\rm{\it{prev}}}_{i,{\rm{year}}}^{l} - {\rm{\it{prev}}}_{i,{\rm{year}} - 1}^{l}$$

We then calculated a weighted AROC for each pixel by taking a weighted average across the years, where more recent AROC were given more weight in the average. We defined the weights to be:

$$w_{{\rm{year}}} = \frac{{\left({{\rm{\it{year}}} - 2000} \right)^\gamma }}{{\mathop {\sum }\nolimits_{2001}^{2017} \left({{\rm{\it{year}}} - 2000} \right)^\gamma }}$$

where *γ* may be chosen to give varying amounts of weight across the years. For this set of projections, we selected *γ* = 1, resulting in a linear weighting scheme that has been tested and vetted for use in projecting the health-related Sustainable Development Goal)^56^. For any grid cell, we then calculated the weighted AROC to be:

$${\rm{\it{AROC}}}_i = \mathop {\sum}\nolimits_{2001}^{2017} {w_{{\rm{\it{year}}}}{\rm{\it{AROC}}}_{i,{\rm{year}}}^l}$$

Finally, we calculated the projections by applying the weighted AROC at each grid cell to our 2017 posterior mean prevalence:

$${\rm{\it{Proj}}}_{i,2025} = {\rm{logit}}^{ - 1}\left({{\rm{\it{prev}}}_{i,2017}^l + {\rm{\it{AROC}}}_{i,j} \times 8} \right)$$

We used the same process to project country- and administrative-level AROC. This projection scheme was analogous to the methods used in the GBD 2017 measurement of progress and projected attainment of health-related Sustainable Development Goals^56^.

### Limitations

#### Data availability

This work should be assessed in full acknowledgment of the data and methodological limitations. Most importantly, the accuracy of our estimates is critically dependent on the quantity and quality of the underlying data. The availability of relevant data varied both spatially and temporally across Africa (Extended Data Fig. 4), and the lack of relevant data is one of the main sources of uncertainty around our estimates (as seen in Fig. 1f). We have constructed a large database of geo-located EBF prevalence data for the purposes of this analysis; nonetheless, important gaps in data coverage—both spatial and temporal—remain. More local data are necessary to monitor health outcomes and guide quality improvement efforts, and to increase the certainty of our results. Collecting local data from all communities every year would be an insurmountable task for most countries; this study aids in filling the current knowledge gap by producing estimates for areas without data collection based on learned patterns from well-surveyed areas, using the same estimation methods for all areas for comparable results across communities.

#### Data accuracy

In addition, there are several factors related to data quality that should be acknowledged. Data in our analyses were obtained from caregivers of infants at any time point between birth and 6 months of age. Although an infant’s EBF status was based on a single time point (the 24 h preceding the survey interview), which is known to overestimate EBF practice for the full 6-month period, as infants may be fed other foods and liquids either before or after the survey, this estimation is standard practice^57,58^. Following the standard approach for estimating EBF based on international guidelines^57,58^, the proportion of infants who are exclusively breastfed for the full 6 months is calculated by estimating the prevalence of EBF for all children under 6 months of age (though EBF is known to decline with age)^57^. Due to the age range (0- to 5-month-old infants) relevant to the purpose of estimating EBF prevalence, our sample sizes are relatively smaller than previous efforts mapping localized estimates for health conditions, outcomes and socioeconomic indicators^12,13,41,42^, further contributing to the relatively large degree of uncertainty associated with our estimates.

The location information associated with the data compiled for these analyses is subject to some error. To protect respondents’ confidentiality, most surveys that collect GPS coordinates perform some type of random displacement on those coordinates before releasing data for secondary analyses. For example, GPS coordinates for DHS data are displaced by up to 2 km for urban clusters, up to 5 km for most rural clusters, and up to 10 km in a random 1% of rural clusters^59^. Furthermore, data associated with polygons rather than GPS coordinates were resampled so that they could be included in the geostatistical model, but this process essentially assumes that EBF prevalence is constant over the polygon. Research on scalable methods for better integration of polygon data in geostatistical models similar to those used in this analysis is currently ongoing.

#### Modeling limitations

With respect to the modeling strategy, the primary limitation is the difficulty in assessing model performance at the grid cell level. We used cross-validation to assess model performance but, due to the substantial impact of sampling error on estimates derived from single survey clusters, it was necessary to aggregate both the data and predictions when assessing error. Additionally, while we attempted to propagate uncertainty from various sources through the different modeling stages, there are some sources of uncertainty that have not been propagated. In particular, it was not computationally feasible to propagate uncertainty from the submodels in stacking through the geostatistical model. Similarly, although the WorldPop population raster is also composed of estimates associated with some uncertainty, this uncertainty is difficult to quantify and not currently reported, and so we were unable to propagate this uncertainty into our estimates of EBF prevalence for administrative subdivisions that were created using population-weighted averages of grid cell estimates.

Model fitting was carried out using an integrated nested Laplace approximation to the posterior distribution, as implemented in the R-INLA package^49^. Prediction from fitted models was subsequently carried out using the inla.posterior.sample() function, which generates samples from the approximated posterior of the fitted model. Both model fitting and prediction thus require approximations, and these approximations may introduce error. While it is difficult to assess the impact of these approximations in this particular use case, our validation analyses found that our final model has low bias and good coverage of the 95% prediction intervals, which provides some reassurance that the approximation method used, as well as other potential sources of error, are not resulting in appreciable bias or poorly described uncertainty in our reported estimates.

Furthermore, our projection methods are derived from the previous spatiotemporal historical trends and based on the assumption that recent trends will continue; thus, we are not projecting underlying drivers (such as increasing urbanization or changes in population)^60^.

### Reporting Summary

Further information on research design is available in the Nature Research Reporting Summary linked to this article.

## Online content

Any methods, additional references, Nature Research reporting summaries, source data, statements of code and data availability and associated accession codes are available at 10.1038/s41591-019-0525-0.

## Supplementary information

Supplementary Information Supplementary Tables 1–12

Reporting Summary

## Data Availability

The findings of this study are supported by data that are available in public online repositories, data that are publicly available on request from the data provider, and data that are not publicly available due to restrictions by the data provider and which were used under license for the current study (including select data sources in Botswana, Eritrea, Ghana, Kenya, South Africa and Zambia, as indicated in Supplementary Tables 2 and 10). Detailed data sources can be found in Supplementary Tables 2–6 and 10. More information about each data source is available on the GHDx (http://ghdx.healthdata.org/), including information about the data provider and links to where the data can be accessed or requested (where available). Administrative boundaries were retrieved from the Global Administrative Unit Layers dataset, implemented by the FAO within the CountrySTAT and Agricultural Market Information System projects^52^. Land cover was retrieved from the online Data Pool, courtesy of the NASA EOSDIS Land Processes Distributed Active Archive Center, USGS/Earth Resources Observation and Science Center, Sioux Falls, South Dakota^51^. Lakes were retrieved from the Global Lakes and Wetlands Database, courtesy of the World Wildlife Fund and the Center for Environmental Systems Research, University of Kassel^53^. Populations were retrieved from WorldPop^55^. Outputs of these EBF analyses at national, administrative and 5 km × 5 km levels throughout Africa are publicly available at the GHDx (http://ghdx.healthdata.org/record/ihme-data/africa-exclusive-breastfeeding-prevalence-geospatial-estimates-2000-2017) and can be explored through our customized visualization tools (https://vizhub.healthdata.org/lbd/ebf). EBF estimates, at various spatial levels, can be explored using custom online data visualization tools (http://vizhub.healthdata.org/lbd/ebf), and are publicly available at the GHDx (http://ghdx.healthdata.org/record/ihme-data/africa-exclusive-breastfeeding-prevalence-geospatial-estimates-2000-2017). The data that support the findings of this study are available on the GHDx; however, some of these data were used under licenses for the current study and are not publically available. All data sources are indicated in Supplementary Table 2, and data with restrictions are indicated with an obelisk symbol.

## Code availability

Our study follows the Guidelines for Accurate and Transparent Health Estimates Reporting (Supplementary Table 1). All code used for these analyses is publicly available online at https://github.com/ihmeuw/lbd/tree/ebf-africa-2019.
