# Publisher Correction: Mapping exclusive breastfeeding in Africa between 2000 and 2017

**DOI:** 10.1038/s41591-019-0577-1

**Published:** 2019-08-27

**Authors:** Natalia V. Bhattacharjee, Lauren E. Schaeffer, Laurie B. Marczak, Jennifer M. Ross, Scott J. Swartz, James Albright, William M. Gardner, Chloe Shields, Amber Sligar, Megan F. Schipp, Brandon V. Pickering, Nathaniel J. Henry, Kimberly B. Johnson, Celia Louie, Michael A. Cork, Krista M. Steuben, Alice Lazzar-Atwood, Dan Lu, Damaris K. Kinyoki, Aaron Osgood-Zimmerman, Lucas Earl, Jonathan F. Mosser, Aniruddha Deshpande, Roy Burstein, Lauren P. Woyczynski, Katherine F. Wilson, John D. VanderHeide, Kirsten E. Wiens, Robert C. Reiner, Ellen G. Piwoz, Rahul Rawat, Benn Sartorius, Nicole Davis Weaver, Molly R. Nixon, David L. Smith, Nicholas J. Kassebaum, Emmanuela Gakidou, Stephen S. Lim, Ali H. Mokdad, Christopher J. L. Murray, Laura Dwyer-Lindgren, Simon I. Hay

**Affiliations:** 1 Institute for Health Metrics and Evaluation, University of Washington, Seattle, WA USA; 2 Department of Global Health, University of Washington, Seattle, WA USA; 3 Department of Medicine, University of Washington, Seattle, WA USA; 4 Department of Health Metrics Sciences, University of Washington, Seattle, WA USA; 5 Bill & Melinda Gates Foundation, Seattle, WA USA; 6 Faculty of Infectious and Tropical Diseases, London School of Hygiene & Tropical Medicine, London, UK; 7 Department of Anesthesiology and Pain Medicine, University of Washington, Seattle, WA USA

**Keywords:** Public health, Risk factors, Paediatrics

Correction to: *Nature Medicine* 10.1038/s41591-019-0525-0, published online 22 July 2019.

Corrections have been made to the version of this article that was initially published, specifically in the Methods subsections ‘Geostatistical model’ and ‘Sensitivity analyses’. First and foremost, corrections have been made to ensure that in the equations, only vectors were typeset in boldface. Thus, in the subsections ‘Geostatistical model’ (first and fourth display equations) and ‘Sensitivity analyses’ (equations 1 and 2), the term “***Β***_***0***_ ” was corrected to appear as a scalar quantity (in non-boldface) “*Β*_*0*_*”*; and in subsection ‘Geostatistical model’ (first display equation), the non-vector term “ **Σ** ” was corrected to appear as the non-boldface “ Σ,” representing summation. Corrections to bold vectors were also made; in the subsection ‘Geostatistical analysis (first display equation), the term “ ε_GP_,” was corrected to appear as a vector (in boldface) “ **ε**_GP_ ”, and in ‘Sensitivity analyses’ (equations 4 and 5), the terms “*Β*_*raw*_ ” and “*Β*_*stack*_ ” were corrected to appear as vectors (in boldface) “***Β***_***raw***_ ” and “***Β***_***stack***_ ”, respectively.

Additionally, minor corrections to spacing and punctuation were made to ensure the correct interpretation of the equations; in the subsection ‘Geostatistical model’ (first display equation) the space in “logit (*p*_*i*_)” was removed and corrected to “logit(*p*_*i*_)”, (second display equation) the spaces in “1 . 2^2^” were removed and corrected to “1.2^2^”, and (third display equation) the semicolons (“;”) were replaced with commas (“,”).

The errors have been corrected in the HTML and PDF versions of the article.

