# Supplementary material for: Mapping exclusive breastfeeding in Africa between 2000 and 2017
Source: Nat Med. 2019 Jul 22;25(8):1205–12. doi: 10.1038/s41591-019-0525-0 (PMC6749549; doi:10.1038/s41591-019-0525-0)
Supplement: Supplementary file 1 — Supplementary Tables 1–12 [file 41591_2019_525_MOESM1_ESM.pdf]

nature medicine
SUPPLEMENTARY INFORMATION
https://doi.org/10.1038/s41591-019-0525-0
In the format provided by the authors and unedited.

Mapping exclusive breastfeeding in Africa between 2000 and 2017

**Natalia V. Bhattacharjee[1], Lauren E. Schaeffer[1], Laurie B. Marczak[1], Jennifer M. Ross[1,2,3], Scott J. Swartz[3], James Albright[1], William M. Gardner[1], Chloe Shields[1], Amber Sligar[1], Megan F. Schipp[1], Brandon V. Pickering[1], Nathaniel J. Henry[1], Kimberly B. Johnson[1], Celia Louie[1], Michael A. Cork[1], Krista M. Steuben[1], Alice Lazzar-Atwood[1], Dan Lu[1], Damaris K. Kinyoki[1], Aaron Osgood-Zimmerman[1], Lucas Earl[1], Jonathan F. Mosser[1,4], Aniruddha Deshpande[1], Roy Burstein[1], Lauren P. Woyczynski[1], Katherine F. Wilson[1], John D. VanderHeide[1], Kirsten E. Wiens[1], Robert C. Reiner Jr[1,4], Ellen G. Piwoz[5], Rahul Rawat[5], Benn Sartorius[4,6], Nicole Davis Weaver[1], Molly R. Nixon[1], David L. Smith[1,4], Nicholas J. Kassebaum[1,7], Emmanuela Gakidou[1,4], Stephen S. Lim[1,4], Ali H. Mokdad[1,4], Christopher J. L. Murray[1,4], Laura Dwyer-Lindgren[1,4,8] and Simon I. Hay[1,4,8]*

[1]Institute for Health Metrics and Evaluation, University of Washington, Seattle, WA, USA. [2]Department of Global Health, University of Washington, Seattle, WA, USA. [3]Department of Medicine, University of Washington, Seattle, WA, USA. [4]Department of Health Metrics Sciences, University of Washington, Seattle, WA, USA. [5]Bill & Melinda Gates Foundation, Seattle, WA, USA. [6]Faculty of Infectious and Tropical Diseases, London School of Hygiene & Tropical Medicine, London, UK. [7]Department of Anesthesiology and Pain Medicine, University of Washington, Seattle, WA, USA. [8]These authors jointly supervised this work: Laura Dwyer-Lindgren, Simon I. Hay. *e-mail: sihay@uw.edu

Contents

Supplementary Table 1. Compliance with the Guidelines for Accurate and Transparent Health Estimates Reporting (GATHER) 2

Supplementary Table 2. Household survey microdata used in mapping exclusive breastfeeding among infants under 6 months 4

Supplementary Table 3. Household survey reports used in mapping exclusive breastfeeding among infants under 6 months 29

Supplementary Table 4. Data excluded from both the geostatistical model and GBD estimates 31

Supplementary Table 5. Data excluded from GBD estimates but included in geostatistical model 32

Supplementary Table 6. Data excluded from geostatistical model but included in GBD estimates 33

Supplementary Table 7. Sources for covariates used in mapping. 34

Supplementary Table 8. Fitted model parameters 37

Supplementary Table 9. In-sample and out-of-sample validation metrics from the covariate sensitivity analysis by level of aggregation. 38

Supplementary Table 10. List of surveys used in the geostatistical model that are not nationally representative 39

Supplementary Table 11. National and subnational achievement of WHO GNT (2017) 41

Supplementary Table 12. National and subnational achievement of WHO GNT (2025) 41

Supplementary Table 1. Compliance with the Guidelines for Accurate and Transparent Health Estimates Reporting (GATHER)

Item #	Checklist item	Description of Compliance
**Objectives and funding**		
**1**	Define the indicator(s), populations (including age, sex, and geographic entities), and time period(s) for which estimates were made.	Abstract; Main text
**2**	List the funding sources for the work.	Main text: End Notes section
**Data Inputs**		
For all data inputs from multiple sources that are synthesized as part of the study:		
**3**	Describe how the data were identified and how the data were accessed.	Methods: Data extraction and processing section
**4**	Specify the inclusion and exclusion criteria. Identify all ad-hoc exclusions.	Methods: Data extraction and processing section; Supplementary Tables 4–6
**5**	Provide information on all included data sources and their main characteristics. For each data source used, report reference information or contact name/institution, population represented, data collection method, year(s) of data collection, sex and age range, diagnostic criteria or measurement method, and sample size, as relevant.	Extended Data Figure 5; Supplementary Tables 2 and 3
**6**	Identify and describe any categories of input data that have potentially important biases (e.g., based on characteristics listed in item 5).	Methods: Data extraction and processing and Data Accuracy sections
For data inputs that contribute to the analysis but were not synthesized as part of the study:		
**7**	Describe and give sources for any other data inputs.	Supplementary Table 6
For all data inputs:		
**8**	Provide all data inputs in a file format from which data can be efficiently extracted (e.g., a spreadsheet rather than a PDF), including all relevant meta-data listed in item 5. For any data inputs that cannot be shared because of ethical or legal reasons, such as third-party ownership, provide a contact name or the name of the institution that retains the right to the data.	Available through http://ghdx.healthdata.org/record/ihme-data/africa-exclusive-breastfeeding-prevalence-geospatial-estimates-2000-2017
**Data analysis**		
**9**	Provide a conceptual overview of the data analysis method. A diagram may be helpful.	Methods: Data extraction and processing section

**10**	Provide a detailed description of all steps of the analysis, including mathematical formulae. This description should cover, as relevant, data cleaning, data pre-processing, data adjustments and weighting of data sources, and mathematical or statistical model(s).	Methods: Data extraction and processing and Statistical analysis sections
**11**	Describe how candidate models were evaluated and how the final model(s) were selected.	Methods: Statistical analysis section
**12**	Provide the results of an evaluation of model performance, if done, as well as the results of any relevant sensitivity analysis.	Methods: Statistical analysis section
**13**	Describe methods for calculating uncertainty of the estimates. State which sources of uncertainty were, and were not, accounted for in the uncertainty analysis.	Methods: Statistical analysis and Limitations sections
**14**	State how analytic or statistical source code used to generate estimates can be accessed.	Methods: Code Availability section
**Results and Discussion**		
**15**	Provide published estimates in a file format from which data can be efficiently extracted.	Available through http://ghdx.healthdata.org/record/ihme-data/africa-exclusive-breastfeeding-prevalence-geospatial-estimates-2000-2017
**16**	Report a quantitative measure of the uncertainty of the estimates (e.g. uncertainty intervals).	Main text; Methods: Statistical analysis section; Figure 1f
**17**	Interpret results in light of existing evidence. If updating a previous set of estimates, describe the reasons for changes in estimates.	Main text
**18**	Discuss limitations of the estimates. Include a discussion of any modelling assumptions or data limitations that affect interpretation of the estimates.	Methods: Limitations section

Supplementary Table 2. Household survey microdata used in mapping exclusive breastfeeding among infants under 6 months

Country	Year(s)	Name	Geographic detail	Citation	NID♦
Algeria	2012-2013	Algeria Multiple Indicator Cluster Survey 2012-2013	Admin 3	Ministry of Health and Population (Algeria), United Nations Children’s Fund (UNICEF). Algeria Multiple Indicator Cluster Survey 2012-2013. New York, United States: United Nations Children’s Fund (UNICEF), 2016.	210614
Angola	2001	Angola Multiple Indicator Cluster Survey 2001	Admin 1	National Institute of Statistics (Angola), United Nations Children’s Fund (UNICEF). Angola Multiple Indicator Cluster Survey 2001. New York, United States: United Nations Children’s Fund (UNICEF).	687
Angola	2015-2016	Angola Demographic and Health Survey 2015-2016	GPS	ICF International, Ministry of Health (Angola), National Institute of Statistics (Angola), United Nations Children’s Fund (UNICEF). Angola Demographic and Health Survey 2015-2016. Fairfax, United States: ICF International, 2017.	218555
Benin	2001	Benin Demographic and Health Survey 2001	GPS	National Institute of Statistics and Economic Analysis (INSAE) (Benin), ORC Macro. Benin Demographic and Health Survey 2001. Fairfax, United States: ICF International.	18950
Benin	2006	Benin Demographic and Health Survey 2006	Admin 1	Macro International, Inc, National Institute of Statistics and Economic Analysis (INSAE) (Benin), National Program Against AIDS (PNLS) (Benin). Benin Demographic and Health Survey 2006. Fairfax, United States: ICF International.	18959
Benin	2011-2012	Benin Demographic and Health Survey 2011-2012	GPS	ICF International, National Institute of Statistics and Economic Analysis (INSAE) (Benin), National Program Against AIDS (PNLS) (Benin). Benin Demographic and Health Survey 2011-2012. Fairfax, United States: ICF International, 2014.	79839
Benin	2014	Benin Multiple Indicator Cluster Survey 2014	Admin 1	National Institute of Statistics and Economic Analysis (INSAE) (Benin), United Nations Children’s Fund (UNICEF). Benin Multiple Indicator Cluster Survey 2014. New York, United States: United Nations Children’s Fund (UNICEF), 2017.	206075

Country	Year(s)	Name	Geographic detail	Citation	NID[♦]
Botswana	2000	Botswana Multiple Indicator Cluster Survey 2000	Admin 1	Central Statistics Office (Botswana), United Nations Children's Fund (UNICEF). Botswana Multiple Indicator Cluster Survey 2000. New York, United States: United Nations Children's Fund (UNICEF), 2015.	1404
Botswana	2007-2008	Botswana Family Health Survey 2007-2008	Admin 1	Central Statistics Office (Botswana). Botswana Family Health Survey 2007-2008. Gaborone, Botswana: Central Statistics Office (Botswana), 2009.	22125†
Burkina Faso	1998-1999	Burkina Faso Demographic and Health Survey 1998-1999	GPS	Macro International, Inc, National Institute of Statistics and Demography (Burkina Faso). Burkina Faso Demographic and Health Survey 1998-1999. Fairfax, United States: ICF International.	19076
Burkina Faso	2003	Burkina Faso Demographic and Health Survey 2003	GPS	Macro International, Inc, National Institute of Statistics and Demography (Burkina Faso). Burkina Faso Demographic and Health Survey 2003. Fairfax, United States: ICF International.	19088
Burkina Faso	2006	Burkina Faso Multiple Indicator Cluster Survey 2006	Admin 1	National Institute of Statistics and Demography (Burkina Faso), United Nations Children's Fund (UNICEF). Burkina Faso Multiple Indicator Cluster Survey 2006. New York, United States: United Nations Children's Fund (UNICEF).	1927
Burkina Faso	2010-2011	Burkina Faso Demographic and Health Survey 2010-2011	GPS	ICF Macro, Ministry of Health (Burkina Faso), National Institute of Statistics and Demography (Burkina Faso). Burkina Faso Demographic and Health Survey 2010-2011. Fairfax, United States: ICF International.	19133
Burundi	2000	Burundi Multiple Indicator Cluster Survey 2000	Admin 2	Burundi Institute of Statistics and Economic Studies, United Nations Children's Fund (UNICEF). Burundi Multiple Indicator Cluster Survey 2000. New York, United States: United Nations Children's Fund (UNICEF).	1994
Burundi	2010-2011	Burundi Demographic and Health Survey 2010-2011	GPS	Burundi Institute of Statistics and Economic Studies, ICF International, Ministry of Public Health and the Fight Against AIDS (Burundi). Burundi Demographic and Health Survey 2010-2011. Fairfax, United States: ICF International, 2012.	30431

Country	Year(s)	Name	Geographic detail	Citation	NID♦
Burundi	2016-2017	Burundi Demographic and Health Survey 2016-2017	GPS	Burundi Institute of Statistics and Economic Studies, ICF International, Ministry of Public Health and the Fight Against AIDS (Burundi). Burundi Demographic and Health Survey 2016-2017. Fairfax, United States: ICF International, 2018.	286766
Cameroon	1998	Cameroon Demographic and Health Survey 1998	Admin 1	Central Bureau of the Census and Population Studies (Cameroon), Macro International, Inc. Cameroon Demographic and Health Survey 1998. Fairfax, United States: ICF International.	19198
Cameroon	2004	Cameroon Demographic and Health Survey 2004	GPS	Macro International, Inc, National Institute of Statistics (Cameroon). Cameroon Demographic and Health Survey 2004. Fairfax, United States: ICF International.	19211
Cameroon	2006	Cameroon Multiple Indicator Cluster Survey 2006	Admin 1	United Nations Children's Fund (UNICEF), National Institute of Statistics (Cameroon). Cameroon Multiple Indicator Cluster Survey 2006. New York, United States: United Nations Children's Fund (UNICEF).	2063
Cameroon	2011	Cameroon Demographic and Health Survey 2011	GPS	ICF International, Ministry of Economy, Planning and Regional Development (Cameroon), Ministry of Public Health (Cameroon), National Institute of Statistics (Cameroon), Pasteur Center of Cameroon. Cameroon Demographic and Health Survey 2011. Fairfax, United States: ICF International.	19274
Cameroon	2014	Cameroon Multiple Indicator Cluster Survey 2014	Admin 3	Ministry of Public Health (Cameroon), National Institute of Statistics (Cameroon), United Nations Children's Fund (UNICEF). Cameroon Multiple Indicator Cluster Survey 2014. New York, United States: United Nations Children's Fund (UNICEF), 2017.	244455
Central African Republic	2000	Central African Republic Multiple Indicator Cluster Survey 2000	Admin 1	Division of Statistics and Economic Studies (Central African Republic), Ministry of Economy, Planning and International Cooperation (Central African Republic), United Nations Children's Fund (UNICEF). Central African Republic Multiple Indicator Cluster Survey 2000. New York, United States: United Nations Children's Fund (UNICEF).	2209

Country	Year(s)	Name	Geographic detail	Citation	NID♦
Central African Republic	2006	Central African Republic Multiple Indicator Cluster Survey 2006	Admin 1	United Nations Children's Fund (UNICEF). Central African Republic Multiple Indicator Cluster Survey 2006. New York, United States: United Nations Children's Fund (UNICEF).	2223
Central African Republic	2010-2011	Central African Republic Multiple Indicator Cluster Survey 2010-2011	Admin 1	Central African Institute of Statistics, Economic and Social Studies (ICASEES) (Central African Republic), ICF International. Central African Republic Multiple Indicator Cluster Survey 2010-2011. Fairfax, United States: ICF International, 2013.	82832
Chad	2000	Chad Multiple Indicator Cluster Survey 2000	Admin 1	United Nations Children's Fund (UNICEF), Census Bureau (Chad), National Institute of Statistical, Economic and Demographic Studies (Chad). Chad Multiple Indicator Cluster Survey 2000. New York, United States: United Nations Children's Fund (UNICEF).	2244
Chad	2010	Chad Multiple Indicator Cluster Survey 2010	Admin 1	Ministry of Planning, Economy, and International Cooperation (Chad), National Institute of Statistical, Economic and Demographic Studies (Chad), United Nations Children's Fund (UNICEF). Chad Multiple Indicator Cluster Survey 2010. New York, United States: United Nations Children's Fund (UNICEF), 2014.	76701
Chad	2014-2015	Chad Demographic and Health Survey 2014-2015	GPS	ICF International, National Institute of Statistical, Economic and Demographic Studies (Chad). Chad Demographic and Health Survey 2014-2015. Fairfax, United States: ICF International, 2016.	157025
Comoros	2000	Comoros Multiple Indicator Cluster Survey 2000	Admin 1	United Nations Development Programme (UNDP), United Nations Children's Fund (UNICEF). Comoros Multiple Indicator Cluster Survey 2000. New York, United States: United Nations Children's Fund (UNICEF).	3114
Comoros	2012-2013	Comoros Demographic and Health Survey 2012-2013	GPS	General Directorate of Statistics and Forecasting (Comoros), ICF International. Comoros Demographic and Health Survey 2012-2013. Fairfax, United States: ICF International.	76850

Country	Year(s)	Name	Geographic detail	Citation	NID♦
Congo	2011-2012	Congo Demographic and Health Survey 2011-2012	Admin 1	ICF International, Ministry of Health (Congo, Rep.), National Center for Statistics and Economic Studies (Congo, Rep.). Congo Demographic and Health Survey 2011-2012. Fairfax, United States: ICF International.	56151
Côte d'Ivoire	1998-1999	Côte d'Ivoire Demographic and Health Survey 1998-1999	GPS	Macro International, Inc, National Institute of Statistics (Côte d'Ivoire). Côte d'Ivoire Demographic and Health Survey 1998-1999. Calverton, United States: Macro International, Inc.	18531
Côte d'Ivoire	2000	Côte d'Ivoire Multiple Indicator Cluster Survey 2000	Admin 1	National School for Statistics and Economics Applied (ENSEA), United Nations Children's Fund (UNICEF), United Nations Educational, Scientific and Cultural Organization (UNESCO). Côte d'Ivoire Multiple Indicator Cluster Survey 2000. New York, United States: United Nations Children's Fund (UNICEF).	26444
Côte d'Ivoire	2006	Côte d'Ivoire Multiple Indicator Cluster Survey 2006	Admin 3	United Nations Children's Fund (UNICEF), National Institute of Statistics (Côte d'Ivoire). Côte d'Ivoire Multiple Indicator Cluster Survey 2006. New York, United States: United Nations Children's Fund (UNICEF).	26433
Côte d'Ivoire	2011-2012	Côte d'Ivoire Demographic and Health Survey 2011-2012	GPS	ICF International, Ministry of the Fight Against AIDS (Côte d'Ivoire), National Institute of Statistics (Côte d'Ivoire). Côte d'Ivoire Demographic and Health Survey 2011-2012. Fairfax, United States: ICF International.	18533
Côte d'Ivoire	2016	Côte d'Ivoire Multiple Indicator Cluster Survey 2016	Admin 1	National Institute of Statistics (Côte d'Ivoire), United Nations Children's Fund (UNICEF). Cote d'Ivoire Multiple Indicator Cluster Survey 2016. New York, United States: United Nations Children's Fund (UNICEF), 2018.	218611
Democratic Republic of the Congo	2001	Democratic Republic of the Congo Multiple Indicator Cluster Survey 2001	Admin 1	Ministry of Planning and Reconstruction (Congo, DR), United Nations Children's Fund (UNICEF). Congo, DR Multiple Indicator Cluster Survey 2001. New York, United States: United Nations Children's Fund (UNICEF).	3161

Country	Year(s)	Name	Geographic detail	Citation	NID♦
Democratic Republic of the Congo	2007	Democratic Republic of the Congo Demographic and Health Survey 2007	GPS	Macro International, Inc, Ministry of Planning (Congo, DR). Democratic Republic of the Congo Demographic and Health Survey 2007. Fairfax, United States: ICF International.	19381
Democratic Republic of the Congo	2010	Democratic Republic of the Congo Multiple Indicator Cluster Survey 2010	GPS	National Statistical Institute (Congo, DR), Ministry of Planning (Congo, DR), United Nations Children's Fund (UNICEF). Congo, DR Multiple Indicator Cluster Survey 2010. New York, United States: United Nations Children's Fund (UNICEF).	26998
Democratic Republic of the Congo	2013-2014	Democratic Republic of the Congo Demographic and Health Survey 2013-2014	GPS	ICF International, Ministry of Planning and Monitoring Implementation of the Revolution of Modernity (Congo, DR), Ministry of Public Health (Congo, DR), National Institute of Statistics (Congo, DR). Democratic Republic of the Congo Demographic and Health Survey 2013-2014. Fairfax, United States: ICF International, 2014.	76878
Egypt	2000	Egypt Demographic and Health Survey 2000	GPS	Macro International, Inc, Population Council (Egypt). Egypt Demographic and Health Survey 2000. Fairfax, United States: ICF International.	19511
Egypt	2003	Egypt Interim Demographic and Health Survey 2003	GPS	El-Zanaty and Associates, Macro International, Inc, Ministry of Health and Population (Egypt), Population Council (Egypt). Egypt Interim Demographic and Health Survey 2003. Fairfax, United States: ICF International.	19529
Egypt	2008	Egypt Demographic and Health Survey 2008	GPS	El-Zanaty and Associates, Macro International, Inc, Ministry of Health and Population (Egypt). Egypt Demographic and Health Survey 2008. Fairfax, United States: ICF International.	26842
Egypt	2013-2014	Egypt IPHN Rural Districts Multiple Indicator Cluster Survey 2013-2014	Admin 1	El-Zanaty and Associates, Ministry of Health and Population (Egypt), United Nations Children's Fund (UNICEF). Egypt IPHN Rural Districts Multiple Indicator Cluster Survey 2013-2014. New York, United States: United Nations Children's Fund (UNICEF), 2016.	159617
Egypt	2014	Egypt Demographic and Health Survey 2014	GPS	El-Zanaty and Associates, ICF International, Ministry of Health and Population (Egypt). Egypt Demographic and Health Survey 2014. Fairfax, United States: ICF International.	154897

Country	Year(s)	Name	Geographic detail	Citation	NID♦
Equatorial Guinea	2000	Equatorial Guinea Multiple Indicator Cluster Survey 2000	Admin 1	Ministry of Planning, Economic Development and Public Investment (Equatorial Guinea), United Nations Children's Fund (UNICEF). Equatorial Guinea Multiple Indicator Cluster Survey 2000. New York, United States: United Nations Children's Fund (UNICEF).	3655
Eritrea	2002	Eritrea Demographic and Health Survey 2002	Admin 1	Macro International, Inc, National Statistics and Evaluation Office (Eritrea). Eritrea Demographic and Health Survey 2002. Calverton, United States: Macro International, Inc.	19539†
Ethiopia	2000	Ethiopia Demographic and Health Survey 2000	GPS	Central Statistical Agency (Ethiopia), ORC Macro. Ethiopia Demographic and Health Survey 2000. Calverton, United States: ORC Macro, 2001.	19571
Ethiopia	2010-2011	Ethiopia Demographic and Health Survey 2010-2011	GPS	Central Statistical Agency (Ethiopia), ICF Macro, Ministry of Health (Ethiopia). Ethiopia Demographic and Health Survey 2010-2011. Fairfax, United States: ICF International.	21301
Ethiopia	2016	Ethiopia Demographic and Health Survey 2016	GPS	Central Statistical Agency (Ethiopia), ICF International. Ethiopia Demographic and Health Survey 2016. Fairfax, United States: ICF International, 2017.	218568
Gabon	2000-2001	Gabon Demographic and Health Survey 2000-2001	Admin 2	General Directorate of Statistics and Economic Studies (Gabon), Macro International, Inc. Gabon Demographic and Health Survey 2000-2001. Calverton, United States: Macro International, Inc.	19579
Gabon	2012	Gabon Demographic and Health Survey 2012	GPS	General Directorate of Statistics (Gabon), ICF International, Ministry of Economy, Employment and Sustainable Development (Gabon), Ministry of Health (Gabon). Gabon Demographic and Health Survey 2012. Fairfax, United States: ICF International, 2013.	76706
Ghana	1998-1999	Ghana Demographic and Health Survey 1998-1999	GPS	Ghana Statistical Service, Macro International, Inc. Ghana Demographic and Health Survey 1998-1999. Calverton, United States: Macro International, Inc.	19614

Country	Year(s)	Name	Geographic detail	Citation	NID♦
Ghana	2003	Ghana Demographic and Health Survey 2003	GPS	Ghana Statistical Service, Macro International, Inc. Ghana Demographic and Health Survey 2003. Calverton, United States: Macro International, Inc.	19627
Ghana	2006	Ghana Multiple Indicator Cluster Survey 2006	Admin 1	Ministry of Health (MOH) (Ghana), Ghana Statistical Service and United Nations Children's Fund (UNICEF). Ghana Multiple Indicator Cluster Survey 2006. New York, United States: United Nations Children's Fund (UNICEF).	4694
Ghana	2007-2008	Ghana District Multiple Indicator Cluster Survey 2007-2008	Admin 1	Ghana Statistical Service, Ministry of Health (Ghana), United Nations Children's Fund (UNICEF). Ghana District Multiple Indicator Cluster Survey 2007-2008.	160576†
Ghana	2008	Ghana Demographic and Health Survey 2008	GPS	Ghana Statistical Service, Macro International, Inc, Ministry of Health (Ghana). Ghana Demographic and Health Survey 2008. Calverton, United States: Macro International, Inc.	21188
Ghana	2010-2011	Ghana - Accra Multiple Indicator Cluster Survey 2010-2011	GPS	Institute of Statistical, Social and Economic Research, University of Ghana, United Nations Children's Fund (UNICEF). Ghana - Accra Multiple Indicator Cluster Survey 2010-2011. New York, United States: United Nations Children's Fund (UNICEF), 2014.	56241
Ghana	2011	Ghana Multiple Indicator Cluster Survey 2011	GPS	Centers for Disease Control and Prevention (CDC), Ghana Statistical Service, Government of Japan, ICF Macro, Ministry of Health (Ghana), Navrongo Health Research Centre, USAID, United Nations Children's Fund (UNICEF), United Nations Population Fund (UNFPA). Ghana Multiple Indicator Cluster Survey 2011. New York, United States: United Nations Children's Fund (UNICEF), 2013.	63993
Ghana	2014	Ghana Demographic and Health Survey 2014	GPS	Ghana Health Service, Ghana Statistical Service, ICF International. Ghana Demographic and Health Survey 2014. Fairfax, United States: ICF International, 2015.	157027
Guinea	1999	Guinea Demographic and Health Survey 1999	GPS	Macro International, Inc, National Statistics Directorate (Guinea). Guinea Demographic and Health Survey 1999. Calverton, United States: Macro International, Inc.	19670

Country	Year(s)	Name	Geographic detail	Citation	NID♦
Guinea	2005	Guinea Demographic and Health Survey 2005	GPS	Macro International, Inc, National Statistics Directorate (Guinea). Guinea Demographic and Health Survey 2005. Fairfax, United States: ICF International.	19683
Guinea	2012	Guinea Demographic and Health Survey 2012	GPS	ICF Macro, Ministry of Health and Public Hygiene (Guinea), National Institute of Statistics (Guinea). Guinea Demographic and Health Survey 2012. Calverton, United States: ICF Macro, 2014.	69761
Guinea-Bissau	2006	Guinea-Bissau Multiple Indicator Cluster Survey 2006	Admin 1	United Nations Children's Fund (UNICEF), Government of Guinea-Bissau. Guinea-Bissau Multiple Indicator Cluster Survey 2006. New York, United States: United Nations Children's Fund (UNICEF).	4818
Guinea-Bissau	2014	Guinea-Bissau Multiple Indicator Cluster Survey 2014	Admin 1	National Statistics Institute (Guinea-Bissau), United Nations Children's Fund (UNICEF). Guinea-Bissau Multiple Indicator Cluster Survey 2014. New York, United States: United Nations Children's Fund (UNICEF), 2016.	174049
Kenya	1998	Kenya Demographic and Health Survey 1998	GPS	Central Bureau of Statistics (Kenya), Macro International, Inc, National Council for Population Development (NCPD). Kenya Demographic and Health Survey 1998. Calverton, United States: Macro International, Inc.	20132
Kenya	2000	Kenya Multiple Indicator Cluster Survey 2000	GPS	Central Bureau of Statistics (Kenya), United Nations Children's Fund (UNICEF). Kenya Multiple Indicator Cluster Survey 2000. New York, United States: United Nations Children's Fund (UNICEF).	7387
Kenya	2003	Kenya Demographic and Health Survey 2003	GPS	Centers for Disease Control and Prevention (CDC), Central Bureau of Statistics (Kenya), Macro International, Inc, Ministry of Health (Kenya), National Council for Population and Development (Kenya). Kenya Demographic and Health Survey 2003. Calverton, United States: Macro International, Inc.	20145

Country	Year(s)	Name	Geographic detail	Citation	NID[♦]
Kenya	2008	Kenya - Eastern Province Multiple Indicator Cluster Survey 2008	GPS	Kenya National Bureau of Statistics, United Nations Children's Fund (UNICEF). Kenya - Eastern Province Multiple Indicator Cluster Survey 2008. Nairobi, Kenya: Kenya National Bureau of Statistics.	7401
Kenya	2008-2009	Kenya Demographic and Health Survey 2008-2009	GPS	ICF Macro, Kenya Medical Research Institute (KEMRI), Kenya National Bureau of Statistics, Ministry of Public Health and Sanitation (Kenya), National AIDS and STI Control Program (Kenya), National Aids Control Council (NACC), National Coordinating Agency for Population and Development (Kenya). Kenya Demographic and Health Survey 2008-2009. Calverton, United States: ICF Macro.	21365
Kenya	2009	Kenya - Coast Multiple Indicator Cluster Survey 2009	Admin 1	Kenya National Bureau of Statistics, United Nations Children's Fund (UNICEF). Kenya - Coast Multiple Indicator Cluster Survey 2009. New York, United States: United Nations Children's Fund (UNICEF), 2014.	56420
Kenya	2011	Kenya - Nyanza Province Multiple Indicator Cluster Survey 2011	GPS	Kenya National Bureau of Statistics, United Nations Children's Fund (UNICEF). Kenya - Nyanza Province Multiple Indicator Cluster Survey 2011. Nairobi, Kenya: Kenya National Bureau of Statistics.	135416
Kenya	2013-2014	Kenya - Bungoma County Multiple Indicator Survey 2013-2014	GPS	Kenya National Bureau of Statistics, Population Studies and Research Institute, University of Nairobi (Kenya), United Nations Children's Fund (UNICEF). Kenya - Bungoma County Multiple Indicator Survey 2013-2014. New York, United States: United Nations Children's Fund (UNICEF), 2015.	203654†
Kenya	2013-2014	Kenya - Kakamega County Multiple Indicator Survey 2013-2014	GPS	Kenya National Bureau of Statistics, Population Studies and Research Institute, University of Nairobi (Kenya), United Nations Children's Fund (UNICEF). Kenya - Kakamega County Multiple Indicator Survey 2013-2014. New York, United States: United Nations Children's Fund (UNICEF), 2015.	203663†

Country	Year(s)	Name	Geographic detail	Citation	NID♦
Kenya	2013-2014	Kenya - Turkana County Multiple Indicator Survey 2013-2014	GPS	Kenya National Bureau of Statistics, Population Studies and Research Institute, University of Nairobi (Kenya), United Nations Children's Fund (UNICEF). Kenya - Turkana County Multiple Indicator Survey 2013-2014. New York, United States: United Nations Children's Fund (UNICEF), 2015.	203664†
Kenya	2014	Kenya Demographic and Health Survey 2014	GPS	ICF International, Kenya Medical Research Institute (KEMRI), Kenya National Bureau of Statistics, Ministry of Health (Kenya), National AIDS Control Council (Kenya), National Council for Population and Development (Kenya). Kenya Demographic and Health Survey 2014. Fairfax, United States: ICF International.	157057
Lesotho	2004-2005	Lesotho Demographic and Health Survey 2004-2005	GPS	Bureau of Statistics (Lesotho), Macro International, Inc, Ministry of Health and Social Welfare (Lesotho). Lesotho Demographic and Health Survey 2004-2005. Calverton, United States: Macro International, Inc.	20167
Lesotho	2009-2010	Lesotho Demographic and Health Survey 2009-2010	GPS	ICF Macro, Ministry of Health and Social Welfare (Lesotho). Lesotho Demographic and Health Survey 2009-2010. Calverton, United States: ICF Macro.	21382
Lesotho	2014	Lesotho Demographic and Health Survey 2014	GPS	ICF International, Ministry of Health and Social Welfare (Lesotho). Lesotho Demographic and Health Survey 2014. Fairfax, United States: ICF International, 2016.	157058
Liberia	2006-2007	Liberia Demographic and Health Survey 2006-2007	GPS	Liberia Institute for Statistics and Geo-information Services (LISGIS), Macro International, Inc. Liberia Demographic and Health Survey 2006-2007. Calverton, United States: Macro International, Inc.	20191
Liberia	2013	Liberia Demographic and Health Survey 2013	GPS	ICF International, Liberia Institute for Statistics and Geo-information Services (LISGIS), National AIDS and STI Control Program (NACP), Ministry of Health and Social Welfare (Liberia). Liberia Demographic and Health Survey 2013.	77385

Country	Year(s)	Name	Geographic detail	Citation	NID♦
Madagascar	2000	Madagascar Multiple Indicator Cluster Survey 2000	Admin 1	National Institute of Statistics (Madagascar), United Nations Children’s Fund (UNICEF). Madagascar Multiple Indicator Cluster Survey 2000. New York, United States: United Nations Children’s Fund (UNICEF).	27020
Madagascar	2003-2004	Madagascar Demographic and Health Survey 2003-2004	Admin 1	Macro International, Inc, National Institute of Statistics (Madagascar). Madagascar Demographic and Health Survey 2003-2004. Calverton, United States: Macro International, Inc.	20223
Madagascar	2008-2009	Madagascar Demographic and Health Survey 2008-2009	GPS	ICF Macro, National Institute of Statistics (Madagascar). Madagascar Demographic and Health Survey 2008-2009. Calverton, United States: ICF Macro, 2010.	21409
Madagascar	2012	Madagascar - South Multiple Indicator Cluster Survey 2012	GPS	National Institute of Statistics (Madagascar), United Nations Children’s Fund (UNICEF). Madagascar - South Multiple Indicator Cluster Survey 2012. New York, United States: United Nations Children’s Fund (UNICEF), 2015.	125594
Malawi	2000	Malawi Demographic and Health Survey 2000	GPS	Macro International, Inc, National Statistical Office of Malawi. Malawi Demographic and Health Survey 2000. Calverton, United States: Macro International, Inc.	20252
Malawi	2004-2005	Malawi Demographic and Health Survey 2004-2005	GPS	Macro International, Inc, National Statistical Office of Malawi. Malawi Demographic and Health Survey 2004-2005. Calverton, United States: Macro International, Inc.	20263
Malawi	2006	Malawi Multiple Indicator Cluster Survey 2006	Admin 2	United Nations Children’s Fund (UNICEF), National Statistics Office (Malawi). Malawi Multiple Indicator Cluster Survey 2006. New York, United States: United Nations Children’s Fund (UNICEF).	7919
Malawi	2010	Malawi Demographic and Health Survey 2010	GPS	ICF Macro, National Statistical Office of Malawi. Malawi Demographic and Health Survey 2010. Calverton, United States: ICF Macro.	21393

Country	Year(s)	Name	Geographic detail	Citation	NID♦
Malawi	2013-2014	Malawi Multiple Indicator Cluster Survey 2013-2014	Admin 2	National Statistical Office of Malawi, United Nations Children’s Fund (UNICEF). Malawi Multiple Indicator Cluster Survey 2013-2014. New York, United States: United Nations Children’s Fund (UNICEF), 2015.	161662
Malawi	2015-2016	Malawi Demographic and Health Survey 2015-2016	GPS	ICF International, Ministry of Health (Malawi), National Statistical Office of Malawi. Malawi Demographic and Health Survey 2015-2016. Fairfax, United States: ICF International, 2017.	218581
Mali	2001	Mali Demographic and Health Survey 2001	GPS	Macro International, Inc, National Directorate of Statistics and Informatics (DNSI) (Mali), Planning and Statistics Unit, Ministry of Health (Mali). Mali Demographic and Health Survey 2001. Calverton, United States: Macro International, Inc.	20315
Mali	2006	Mali Demographic and Health Survey 2006	GPS	Macro International, Inc, Ministry of Health (Mali), National Directorate of Statistics and Informatics (DNSI) (Mali). Mali Demographic and Health Survey 2006. Calverton, United States: Macro International, Inc.	20274
Mali	2009-2010	Mali Multiple Indicator Cluster Survey 2009-2010	Admin 1	Ministry of Health (Mali), National Institute of Statistics (INSTAT) (Mali), United Nations Children’s Fund (UNICEF). Mali Multiple Indicator Cluster Survey 2009-2010. New York, United States: United Nations Children’s Fund (UNICEF), 2017.	270627
Mali	2012-2013	Mali Demographic and Health Survey 2012-2013	GPS	ICF International, INFO-STAT (Mali), Ministry of Health (Mali), National Institute of Statistics (INSTAT) (Mali), Planning and Statistics Unit, Ministry of Health (Mali). Mali Demographic and Health Survey 2012-2013. Fairfax, United States: ICF International, 2014.	77388

Country	Year(s)	Name	Geographic detail	Citation	NID♦
Mali	2015	Mali Multiple Indicator Cluster Survey 2015	Admin 1	Ministry of Health (Mali), Ministry of Planning (Mali), National Institute of Statistics (INSTAT) (Mali), United Nations Children's Fund (UNICEF). Mali Multiple Indicator Cluster Survey 2015. New York, United States: United Nations Children's Fund (UNICEF), 2017.	248224
Mauritania	2007	Mauritania Multiple Indicator Cluster Survey 2007	Admin 3	National Office of Statistics (Mauritania), United Nations Children's Fund (UNICEF). Mauritania Multiple Indicator Cluster Survey 2007. New York, United States: United Nations Children's Fund (UNICEF).	8115
Mauritania	2011	Mauritania Multiple Indicator Cluster Survey 2011	Admin 3	National Office of Statistics (Mauritania), United Nations Children's Fund (UNICEF). Mauritania Multiple Indicator Cluster Survey 2011. New York, United States: United Nations Children's Fund (UNICEF), 2015.	152783
Mauritania	2015	Mauritania Multiple Indicator Cluster Survey 2015	Admin 1	National Office of Statistics (Mauritania), United Nations Children's Fund (UNICEF). Mauritania Multiple Indicator Cluster Survey 2015. New York, United States: United Nations Children's Fund (UNICEF), 2018.	267343
Morocco	2003-2004	Morocco Demographic and Health Survey 2003-2004	GPS	League of Arab States, Macro International, Inc, Ministry of Health (Morocco). Morocco Demographic and Health Survey 2003-2004. Calverton, United States: Macro International, Inc.	20361
Mozambique	2003-2004	Mozambique Demographic and Health Survey 2003-2004	Admin 1	Macro International, Inc, National Institute of Statistics (INE) (Mozambique). Mozambique Demographic and Health Survey 2003-2004. Calverton, United States: Macro International, Inc.	20394
Mozambique	2008-2009	Mozambique Multiple Indicator Cluster Survey 2008-2009	Admin 1	United Nations Children's Fund (UNICEF), National Statistics Institute (Mozambique). Mozambique Multiple Indicator Cluster Survey 2008-2009. New York, United States: United Nations Children's Fund (UNICEF).	27031

Country	Year(s)	Name	Geographic detail	Citation	NID♦
Mozambique	2011	Mozambique Demographic and Health Survey 2011	GPS	ICF Macro, Manhica Health Research Center (CISM), Ministry of Health (Mozambique), National Institute of Statistics (INE) (Mozambique). Mozambique Demographic and Health Survey 2011. Calverton, United States: ICF Macro, 2013.	55975
Namibia	2000	Namibia Demographic and Health Survey 2000	GPS	Macro International, Inc, Ministry of Health and Social Services (Namibia), National Planning Commission (Namibia). Namibia Demographic and Health Survey 2000. Calverton, United States: Macro International, Inc.	20417
Namibia	2006-2007	Namibia Demographic and Health Survey 2006-2007	GPS	Macro International, Inc, Ministry of Health and Social Services (Namibia). Namibia Demographic and Health Survey 2006-2007. Calverton, United States: Macro International, Inc.	20428
Namibia	2013	Namibia Demographic and Health Survey 2013	GPS	ICF International, Ministry of Health and Social Services (Namibia), Namibia Institute of Pathology, Namibia Statistics Agency. Namibia Demographic and Health Survey 2013. Fairfax, United States: ICF International, 2015.	150382
Niger	1998	Niger Demographic and Health Survey 1998	GPS	CARE International, Macro International, Inc. Niger Demographic and Health Survey 1998. Calverton, United States: Macro International, Inc.	20537
Niger	2000	Niger Multiple Indicator Cluster Survey 2000	Admin 2	Government of Niger, Macro International, Inc, United Nations Children's Fund (UNICEF). Niger Multiple Indicator Cluster Survey 2000. New York, United States: United Nations Children's Fund (UNICEF).	9439
Niger	2006	Niger Demographic and Health Survey 2006	Admin 1	Department of Statistics and National Accounts (Niger), Macro International, Inc. Niger Demographic and Health Survey 2006. Calverton, United States: Macro International, Inc.	20499
Niger	2012	Niger Demographic and Health Survey 2012	Admin 1	ICF International, Ministry of Public Health (Niger), National Institute of Statistics (Niger). Niger Demographic and Health Survey 2012. Fairfax, United States: ICF International, 2014.	74393

Country	Year(s)	Name	Geographic detail	Citation	NID♦
Nigeria	1999	Nigeria Multiple Indicator Cluster Survey 1999	Admin 1	National Bureau of Statistics (Nigeria), United Nations Children's Fund (UNICEF). Nigeria Multiple Indicator Cluster Survey 1999. Abuja, Nigeria: National Bureau of Statistics (Nigeria).	9506
Nigeria	2003	Nigeria Demographic and Health Survey 2003	GPS	Department for International Development (DFiD) (United Kingdom), National Population Commission of Nigeria, ORC Macro, United Nations Children’s Fund (UNICEF), United Nations Population Fund (UNFPA). Nigeria Demographic and Health Survey 2003. Calverton, United States: ORC Macro.	20567
Nigeria	2008	Nigeria Demographic and Health Survey 2008	GPS	Macro International, Inc, National Population Commission of Nigeria. Nigeria Demographic and Health Survey 2008. Calverton, United States: Macro International, Inc, 2009.	21433
Nigeria	2011	Nigeria Multiple Indicator Cluster Survey 2011	Admin 1	National Bureau of Statistics (Nigeria), United Nations Children’s Fund (UNICEF). Nigeria Multiple Indicator Cluster Survey 2011. New York, United States: United Nations Children’s Fund (UNICEF), 2013.	76703
Nigeria	2013	Nigeria Demographic and Health Survey 2013	GPS	ICF International, National Population Commission of Nigeria. Nigeria Demographic and Health Survey 2013. Fairfax, United States: ICF International, 2014.	77390
Nigeria	2016-2017	Nigeria Multiple Indicator Cluster Survey with National Immunization Coverage Survey Supplement 2016-2017	GPS	National Agency for the Control of AIDS (Nigeria), National Bureau of Statistics (Nigeria), National Primary Health Care Development Agency (NPHCDA) (Nigeria), United Nations Children’s Fund (UNICEF). Nigeria Multiple Indicator Cluster Survey with National Immunization Coverage Survey Supplement 2016-2017. New York, United States: United Nations Children’s Fund (UNICEF), 2018.	218613
Rwanda	2000	Rwanda Demographic and Health Survey 2000	Admin 1	Macro International, Inc, National Office of Population (Rwanda). Rwanda Demographic and Health Survey 2000. Calverton, United States: Macro International, Inc.	20722

Country	Year(s)	Name	Geographic detail	Citation	NID♦
Rwanda	2000	Rwanda Multiple Indicator Cluster Survey 2000	Admin 1	Department of Statistics (Rwanda), United Nations Children's Fund (UNICEF). Rwanda Multiple Indicator Cluster Survey 2000. New York, United States: United Nations Children's Fund (UNICEF).	26930
Rwanda	2005	Rwanda Demographic and Health Survey 2005	GPS	Macro International, Inc, National Institute of Statistics of Rwanda. Rwanda Demographic and Health Survey 2005. Calverton, United States: Macro International, Inc.	20740
Rwanda	2010-2011	Rwanda Demographic and Health Survey 2010-2011	GPS	ICF Macro, Ministry of Health (Rwanda), National Institute of Statistics of Rwanda. Rwanda Demographic and Health Survey 2010-2011. Calverton, United States: ICF Macro.	56040
Rwanda	2014-2015	Rwanda Demographic and Health Survey 2014-2015	GPS	ICF International, Ministry of Health (Rwanda), National Institute of Statistics of Rwanda. Rwanda Demographic and Health Survey 2014-2015. Fairfax, United States: ICF International, 2016.	157063
São Tomé and Príncipe	2000	Multiple Indicator Cluster Survey 2000	Admin 1	National Institute of Statistics (), United Nations Children's Fund (UNICEF). São Tomé and Príncipe Multiple Indicator Cluster Survey 2000. New York, United States: United Nations Children's Fund (UNICEF).	27055
São Tomé and Príncipe	2008-2009	Demographic and Health Survey 2008-2009	Admin 2	ICF Macro, Ministry of Health (), National Institute of Statistics (). Demographic and Health Survey 2008-2009. Calverton, United States: ICF Macro.	26866
São Tomé and Príncipe	2014	São Tomé and Príncipe Multiple Indicator Cluster Survey 2014	Admin 1	Global Fund to Fight AIDS, Tuberculosis and Malaria (GFATM), ICF International, National Center for Endemic Diseases (CNE) (), National Institute of Statistics (), United Nations Children's Fund (UNICEF), United Nations Development Programme (UNDP). Multiple Indicator Cluster Survey 2014. New York, United States: United Nations Children's Fund (UNICEF), 2016.	214640

Country	Year(s)	Name	Geographic detail	Citation	NID[♦]
Senegal	2005	Senegal Demographic and Health Survey 2005	GPS	Ministry of Health and Prevention (Senegal), Research Center for Human Development (Senegal). Senegal Demographic and Health Survey 2005. Calverton, United States: Macro International, Inc.	26855
Senegal	2010-2011	Senegal Demographic and Health Survey 2010-2011	GPS	Center for Research in Human Development (CRDH), Cheikh Anta Diop University, Hospital Aristide Le Dantec, ICF Macro, National Agency of Statistics and Demography (Senegal). Senegal Demographic and Health Survey 2010-2011. Calverton, United States: ICF Macro.	56063
Senegal	2012-2013	Senegal Continuous Demographic and Health Survey 2012-2013	GPS	ICF International, Ministry of Health and Social Action (Senegal), National Agency of Statistics and Demography (Senegal). Senegal Continuous Demographic and Health Survey 2012-2013. Fairfax, United States: ICF International, 2014.	111432
Senegal	2014	Senegal Continuous Demographic and Health Survey 2014	Admin 1	Cheikh Anta Diop University, ICF International, National Agency of Statistics and Demography (Senegal). Senegal Continuous Demographic and Health Survey 2014. Fairfax, United States: ICF International, 2015.	191270
Senegal	2015	Senegal Continuous Demographic and Health Survey 2015	GPS	Cheikh Anta Diop University, ICF International, National Agency of Statistics and Demography (Senegal). Senegal Continuous Demographic and Health Survey 2015. Fairfax, United States: ICF International, 2016.	218592
Senegal	2016	Senegal Continuous Demographic and Health Survey 2016	GPS	ICF International, Ministry of Health and Social Action (Senegal), National Agency of Statistics and Demography (Senegal). Senegal Continuous Demographic and Health Survey 2016. Fairfax, United States: ICF International, 2017.	286772
Senegal*	2017	Senegal Continuous Demographic and Health Survey 2017	Admin 1	ICF International, Ministry of Health and Social Action (Senegal), National Agency of Statistics and Demography (Senegal), Unit for the Fight Against Malnutrition (Senegal). Senegal Continuous Demographic and Health Survey 2017. Fairfax, United States: ICF International, 2018.	353526

Country	Year(s)	Name	Geographic detail	Citation	NID♦
Sierra Leone	2000	Sierra Leone Multiple Indicator Cluster Survey 2000	Admin 1	Central Statistics Office (Sierra Leone), United Nations Children's Fund (UNICEF). Sierra Leone Multiple Indicator Cluster Survey 2000. New York, United States: United Nations Children's Fund (UNICEF).	11639
Sierra Leone	2005	Sierra Leone Multiple Indicator Cluster Survey 2005	Admin 2	United Nations Children's Fund (UNICEF), Statistics Sierra Leone. Sierra Leone Multiple Indicator Cluster Survey 2005. New York, United States: United Nations Children's Fund (UNICEF).	11649
Sierra Leone	2008	Sierra Leone Demographic and Health Survey 2008	GPS	Macro International, Inc, Statistics Sierra Leone. Sierra Leone Demographic and Health Survey 2008. Calverton, United States: Macro International, Inc.	21258
Sierra Leone	2010	Sierra Leone Multiple Indicator Cluster Survey 2010	Admin 2	Statistics Sierra Leone, United Nations Children's Fund (UNICEF). Sierra Leone Multiple Indicator Cluster Survey 2010. New York, United States: United Nations Children's Fund (UNICEF).	76700
Sierra Leone	2013	Sierra Leone Demographic and Health Survey 2013	GPS	ICF International, Ministry of Health and Sanitation (Sierra Leone), Statistics Sierra Leone. Sierra Leone Demographic and Health Survey 2013. Fairfax, United States: ICF International, 2014.	131467
Sierra Leone*	2017	Sierra Leone Multiple Indicator Cluster Survey 2017	Admin 2	Statistics Sierra Leone, United Nations Children's Fund (UNICEF). Sierra Leone Multiple Indicator Cluster Survey 2017. New York, United States: United Nations Children's Fund (UNICEF), 2018.	218619
Somalia	2006	Somalia Multiple Indicator Cluster Survey 2006	Admin 2	Pan Arab Project for Family Health (PAPFAM), United Nations Children's Fund (UNICEF). Somalia Multiple Indicator Cluster Survey 2006. New York, United States: United Nations Children's Fund (UNICEF).	11774

Country	Year(s)	Name	Geographic detail	Citation	NID♦
Somalia	2011	Somalia - Northeast Zone Multiple Indicator Cluster Survey 2011	GPS	Puntland Ministry of Planning and International Cooperation (Somalia), United Nations Children's Fund (UNICEF). Somalia - Northeast Zone Multiple Indicator Cluster Survey 2011. New York, United States: United Nations Children's Fund (UNICEF), 2015.	91508
Somalia	2011	Somalia - Somaliland Multiple Indicator Cluster Survey 2011	GPS	Ministry of National Planning and Development (Somaliland), United Nations Children's Fund (UNICEF). Somalia - Somaliland Multiple Indicator Cluster Survey 2011. New York, United States: United Nations Children's Fund (UNICEF), 2015.	91507
South Africa	1998	South Africa Demographic and Health Survey 1998	Admin 2	Department of Health (South Africa), Macro International, Inc, South African Medical Research Council. South Africa Demographic and Health Survey 1998. Calverton, United States: Macro International, Inc.	20796
South Africa*	2016	South Africa Demographic and Health Survey 2016	GPS	Department of Health (South Africa), ICF International, South African Medical Research Council, Statistics South Africa. South Africa Demographic and Health Survey 2016. Fairfax, United States: ICF International, 2019.	157064
Sudan	1999	Sudan - South Multiple Indicator Cluster Survey 1999	Admin 1	United Nations Children's Fund (UNICEF). South Sudan Multiple Indicator Cluster Survey 1999. New York, United States: United Nations Children's Fund (UNICEF).	12232
Sudan	2000	Sudan Multiple Indicator Cluster Survey 2000	Admin 1	Central Bureau of Statistics (Sudan), Federal Ministry of Health (Sudan), United Nations Children's Fund (UNICEF). Sudan Multiple Indicator Cluster Survey 2000. New York, United States: United Nations Children's Fund (UNICEF).	12243
Sudan	2010	Sudan - North Multiple Indicator Cluster Survey 2010	Admin 1	Central Bureau of Statistics (Sudan), Ministry of Health (South Sudan). Sudan - North Multiple Indicator Cluster Survey 2010. New York, United States: United Nations Children's Fund (UNICEF), 2015.	153643

Country	Year(s)	Name	Geographic detail	Citation	NID[♦]
Sudan	2010	Sudan - South Multiple Indicator Cluster Survey 2010	Admin 4	Central Bureau of Statistics (Sudan), Federal Ministry of Health (Sudan), Government of Sudan, Ministry of Health (South Sudan), Southern Sudan Centre for Census, Statistics and Evaluation. Sudan - South Multiple Indicator Cluster Survey 2010. New York, United States: United Nations Children's Fund (UNICEF), 2015.	32189
Sudan	2014	Sudan Multiple Indicator Cluster Survey 2014	Admin 1	Central Bureau of Statistics (Sudan), Federal Ministry of Health (Sudan), United Nations Children's Fund (UNICEF). Sudan Multiple Indicator Cluster Survey 2014. New York, United States: United Nations Children's Fund (UNICEF), 2016.	200617
Swaziland (eSwatini)	2000	Swaziland Multiple Indicator Cluster Survey 2000	Admin 1	Central Statistical Office (Swaziland), United Nations Children's Fund (UNICEF). Swaziland Multiple Indicator Cluster Survey 2000. New York, United States: United Nations Children's Fund (UNICEF).	12320
Swaziland (eSwatini)	2006-2007	Swaziland Demographic and Health Survey 2006-2007	GPS	Central Statistical Office (Swaziland), Macro International, Inc. Swaziland Demographic and Health Survey 2006-2007. Calverton, United States: Macro International, Inc.	20829
Swaziland (eSwatini)	2010	Swaziland Multiple Indicator Cluster Survey 2010	Admin 1	Central Statistical Office (Swaziland), United Nations Children's Fund (UNICEF). Swaziland Multiple Indicator Cluster Survey 2010. New York, United States: United Nations Children's Fund (UNICEF).	30325
Swaziland (eSwatini)	2014	Swaziland Multiple Indicator Cluster Survey 2014	Admin 1	Central Statistical Office (Swaziland), United Nations Children's Fund (UNICEF), United Nations Educational, Scientific and Cultural Organization (UNESCO), United Nations Population Fund (UNFPA). Swaziland Multiple Indicator Cluster Survey 2014. New York, United States: United Nations Children's Fund (UNICEF), 2016.	200707
Tanzania	1999	Tanzania Demographic and Health Survey 1999	GPS	Macro International, Inc, National Bureau of Statistics (Tanzania). Tanzania Demographic and Health Survey 1999. Calverton, United States: Macro International, Inc.	20865

Country	Year(s)	Name	Geographic detail	Citation	NID[♦]
Tanzania	2004-2005	Tanzania Demographic and Health Survey 2004-2005	Admin 4	Macro International, Inc, National Bureau of Statistics (Tanzania). Tanzania Demographic and Health Survey 2004-2005. Calverton, United States: Macro International, Inc.	20875
Tanzania	2009-2010	Tanzania Demographic and Health Survey 2009-2010	GPS	ICF Macro, National Bureau of Statistics (Tanzania). Tanzania Demographic and Health Survey 2009-2010. Calverton, United States: ICF Macro.	21331
Tanzania	2015-2016	Tanzania Demographic and Health Survey 2015-2016	GPS	ICF International, Ministry of Health (Zanzibar), Ministry of Health, Community Development, Gender, Elderly and Children (MoHCDEC) (Tanzania), National Bureau of Statistics (Tanzania), Office of Chief Government Statistician (OCGS-Zanzibar). Tanzania Demographic and Health Survey 2015-2016. Fairfax, United States: ICF International, 2016.	218593
The Gambia	2000	Gambia Multiple Indicator Cluster Survey 2000	Admin 1	Central Statistics Department (Gambia), United Nations Children's Fund (UNICEF). Gambia Multiple Indicator Cluster Survey 2000. New York, United States: United Nations Children's Fund (UNICEF).	3922
The Gambia	2005-2006	Gambia Multiple Indicator Cluster Survey 2005-2006	Admin 2	Gambia Bureau of Statistics (GBOS), United Nations Children's Fund (UNICEF). Gambia Multiple Indicator Cluster Survey 2005-2006. New York, United States: United Nations Children's Fund (UNICEF).	3935
The Gambia	2010	Gambia Multiple Indicator Cluster Survey 2010	Admin 1	Gambia Bureau of Statistics (GBOS), United Nations Children's Fund (UNICEF). Gambia Multiple Indicator Cluster Survey 2010. New York, United States: United Nations Children's Fund (UNICEF), 2018.	91506
The Gambia	2013	Gambia Demographic and Health Survey 2013	Admin 2	Gambia Bureau of Statistics (GBOS), ICF International, Ministry of Health and Social Welfare (Gambia). Gambia Demographic and Health Survey 2013. Fairfax, United States: ICF International, 2015.	77384
Togo	1998	Togo Demographic and Health Survey 1998	GPS	Department of Statistics (Togo), Macro International, Inc. Togo Demographic and Health Survey 1998. Calverton, United States: Macro International, Inc.	20909

Country	Year(s)	Name	Geographic detail	Citation	NID♦
Togo	2000	Togo Multiple Indicator Cluster Survey 2000	GPS	United Nations Children’s Fund (UNICEF). Togo Multiple Indicator Cluster Survey 2000. New York, United States: United Nations Children’s Fund (UNICEF).	12886
Togo	2006	Togo Multiple Indicator Cluster Survey 2006	Admin 1	Directorate General of Statistics and National Accounting (Togo), United Nations Children’s Fund (UNICEF). Togo Multiple Indicator Cluster Survey 2006. New York, United States: United Nations Children’s Fund (UNICEF).	12896
Togo	2010	Togo Multiple Indicator Cluster Survey 2010	Admin 1	Directorate General of Statistics and National Accounting (Togo), United Nations Children’s Fund (UNICEF). Togo Multiple Indicator Cluster Survey 2010. New York, United States: United Nations Children’s Fund (UNICEF).	40021
Togo	2013-2014	Togo Demographic and Health Survey 2013-2014	Admin 1	Directorate General of Statistics and National Accounts (Togo), ICF International, Ministry of Health (Togo). Togo Demographic and Health Survey 2013-2014. Fairfax, United States: ICF International, 2015.	77515
Tunisia	2011-2012	Tunisia Multiple Indicator Cluster Survey 2011-2012	Admin 1	Ministry of Regional Development and Planning (Tunisia), National Institute of Statistics (Tunisia), United Nations Children’s Fund (UNICEF). Tunisia Multiple Indicator Cluster Survey 2011-2012. New York, United States: United Nations Children’s Fund (UNICEF), 2014.	76709
Uganda	2000-2001	Uganda Demographic and Health Survey 2000-2001	GPS	Macro International, Inc, Uganda Bureau of Statistics. Uganda Demographic and Health Survey 2000-2001. Calverton, United States: Macro International, Inc.	20993
Uganda	2006	Uganda Demographic and Health Survey 2006	GPS	Macro International, Inc, Uganda Bureau of Statistics. Uganda Demographic and Health Survey 2006. Calverton, United States: Macro International, Inc.	21014
Uganda	2011	Uganda Demographic and Health Survey 2011	GPS	ICF Macro, Uganda Bureau of Statistics. Uganda Demographic and Health Survey 2011. Fairfax, United States: ICF International.	56021

Country	Year(s)	Name	Geographic detail	Citation	NID[♦]
Uganda	2016	Uganda Demographic and Health Survey 2016	GPS	ICF International, Uganda Bureau of Statistics. Uganda Demographic and Health Survey 2016. Fairfax, United States: ICF International, 2018.	286780
Zambia	1999	Zambia Multiple Indicator Cluster Survey 1999	Admin 1	Central Statistical Office (Zambia), Food Security, Health and Nutrition Information System (Zambia), United Nations Children's Fund (UNICEF). Zambia Multiple Indicator Cluster Survey 1999. New York, United States: United Nations Children's Fund (UNICEF).	14122
Zambia	2001-2002	Zambia Demographic and Health Survey 2001-2002	Admin 1	Central Board of Health (Zambia), Central Statistical Office (Zambia), Macro International, Inc. Zambia Demographic and Health Survey 2001-2002. Calverton, United States: Macro International, Inc.	21102
Zambia	2002-2003	Zambia Living Conditions Monitoring Survey 2002-2003	Admin 2	Central Statistical Office (Zambia). Zambia Living Conditions Monitoring Survey 2002-2003. Lusaka, Zambia: Central Statistical Office (Zambia).	14027
Zambia	2007	Zambia Demographic and Health Survey 2007	GPS	Central Statistical Office (Zambia), Macro International, Inc. Zambia Demographic and Health Survey 2007. Calverton, United States: Macro International, Inc.	21117
Zambia	2010	Zambia Living Conditions Monitoring Survey 2010	Admin 2	Central Statistical Office (Zambia). Zambia Living Conditions Monitoring Survey 2010.	58660†
Zambia	2013-2014	Zambia Demographic and Health Survey 2013-2014	GPS	Central Statistical Office (Zambia), ICF International, Ministry of Health (Zambia), Tropical Diseases Research Centre, University Teaching Hospital (Zambia), University of Zambia. Zambia Demographic and Health Survey 2013-2014. Fairfax, United States: ICF International, 2015.	77516
Zimbabwe	1999	Zimbabwe Demographic and Health Survey 1999	GPS	Central Statistical Office (Zimbabwe), Macro International, Inc. Zimbabwe Demographic and Health Survey 1999. Calverton, United States: Macro International, Inc.	21151

Country	Year(s)	Name	Geographic detail	Citation	NID♦
Zimbabwe	2005-2006	Zimbabwe Demographic and Health Survey 2005-2006	GPS	Central Statistical Office (Zimbabwe), Macro International, Inc. Zimbabwe Demographic and Health Survey 2005-2006. Calverton, United States: Macro International, Inc.	21163
Zimbabwe	2009	Zimbabwe Multiple Indicator Monitoring Survey 2009	Admin 1	Central Statistical Office (Zimbabwe). Zimbabwe Multiple Indicator Monitoring Survey 2009. New York, United States: United Nations Children's Fund (UNICEF).	35493
Zimbabwe	2010-2011	Zimbabwe Demographic and Health Survey 2010-2011	GPS	ICF Macro, Zimbabwe National Statistics Agency. Zimbabwe Demographic and Health Survey 2010-2011. Calverton, United States: ICF Macro, 2012.	55992
Zimbabwe	2014	Zimbabwe Multiple Indicator Cluster Survey 2014	Admin 1	United Nations Children’s Fund (UNICEF), Zimbabwe National Statistics Agency. Zimbabwe Multiple Indicator Cluster Survey 2014. New York, United States: United Nations Children’s Fund (UNICEF), 2015.	152720
Zimbabwe	2015	Zimbabwe Demographic and Health Survey 2015	GPS	ICF International, National Microbiology Reference Laboratory, Harare Central Hospital (NMRL) (Zimbabwe), Zimbabwe National Statistics Agency. Zimbabwe Demographic and Health Survey 2015. Fairfax, United States: ICF International, 2016.	157066

♦NID = Data source unique identifier in the Global Health Data Exchange (GHDx) (http://ghdx.healthdata.org/). Additional information about each data source is available via the GHDx, including information about the data provider and links to where the data can be accessed or requested (where available). NIDs can be entered in the search bar to retrieve the record for a particular source.

*Since the publication of GBD 2017, additional surveys (South Africa DHS 2016, Senegal DHS 2017 and Sierra Leone MICS 2017) have been released, and we incorporated these into the country-level estimates to which we calibrate our models.

†Data source is not publicly available due to restrictions by the data provider and was used under license for the current study.
All data sources included in this study are cited unless prohibited by original data holder.

Supplementary Table 3. Household survey reports used in mapping exclusive breastfeeding among infants under 6 months

Country	Year(s)	Name	Geographic detail	Citation	NID♦
Algeria*	2006	Algeria Multiple Indicator Cluster Survey 2006	Admin 0.5**	Joint United Nations Program on HIV/AIDS (UNAIDS), Ministry of Health, Population and Hospital Reform (Algeria), National Office of Statistics (Algeria), United Nations Children's Fund (UNICEF), United Nations Population Fund (UNFPA). Algeria Multiple Indicator Cluster Survey 2006.	641
Burkina Faso*	2012	Burkina Faso National Nutrition Survey 2012	Admin 2	Ministry of Health (Burkina Faso), National Institute of Statistics and Demography (Burkina Faso). Burkina Faso National Nutrition Survey 2012.	262834
Burkina Faso*	2016	Burkina Faso National Nutrition Survey 2016	Admin 1	Ministry of Health (Burkina Faso), National Institute of Statistics and Demography (Burkina Faso). Burkina Faso National Nutrition Survey 2016.	299307
Mali*	2016	Mali National Anthropometric Nutrition Survey and Mortality Retrospective June – August 2016	Admin 3	Government of Mali, National Institute of Statistics (INSTAT) (Mali). Mali National Anthropometric Nutrition Survey and Mortality Retrospective June - August 2016.	297069
Niger*	2009	Niger Nutrition and Child Survival Survey	Admin 1	Ministry of Public Health (Niger), National Institute of Statistics (Niger). Niger Nutrition and Child Survival Survey 2009.	160053
São Tomé and Príncipe*	2006	São Tomé and Príncipe Multiple Indicator Cluster Survey 2006	Admin 2	National Institute of Statistics (São Tomé and Príncipe), United Nations Children's Fund (UNICEF). São Tomé and Príncipe Multiple Indicator Cluster Survey 2006. New York, United States: United Nations Children's Fund (UNICEF).	11434

Country	Year(s)	Name	Geographic detail	Citation	NID♦
Somalia*	2009	Somalia National Micronutrient and Anthropometric Survey 2009	Admin 0.5**	Centre for International Health and Development, Institute of Child Health (CIHD), Food and Nutrition Security Analysis Unit (Somalia), Ministry of Health (Somalia), Ministry of Health and Labor (Somaliland), Puntland Ministry of Health (Somalia), United Nations Children's Fund (UNICEF), World Food Programme (WFP), World Health Organization (WHO). Somalia National Micronutrient and Anthropometric Survey 2009.	152968

♦NID = Data source unique identifier in the Global Health Data Exchange (GHDx) (http://ghdx.healthdata.org/). Additional information about each data source is available via the GHDx, including information about the data provider and links to where the data can be accessed or requested (where available). NIDs can be entered in the search bar to retrieve the record for a particular source.

*Since the publication of GBD 2017, additional survey reports were incorporated into the country-level estimates to which we calibrate our models.

**Admin 0.5 refers to subnational data at a lower resolution than Admin 1.

Supplementary Table 4. Data excluded from both the geostatistical model and GBD estimates

Country	Series	Year(s)	Citation	NID♦	Rationale for exclusion
Mali	Multiple Indicator Cluster Survey (MICS)	2009-2010	Ministry of Health (Mali), National Institute of Statistics (INSTAT) (Mali), United Nations Children's Fund (UNICEF). Mali Multiple Indicator Cluster Survey 2009-2010. New York, United States: United Nations Children's Fund (UNICEF), 2017.	270627	Survey estimates are systematically low compared to estimates from other established survey series (2006 DHS, 2012 DHS)
Mali	LSMS	2014-2015	Ministry of Rural Development (Mali), National Institute of Statistics (INSTAT) (Mali), World Bank. Mali Agricultural Integrated Economic Survey 2014-2015. Washington DC, United States: World Bank.	260407	Survey estimates are implausibly high compared to estimates from other established survey series (2012 DHS)
Nigeria	Core Welfare Indicators Questionnaire Survey (CWIQ)	2006-2007	National Bureau of Statistics (Nigeria). Nigeria Core Welfare Indicators Questionnaire Survey 2006. Abuja, Nigeria: National Bureau of Statistics (Nigeria).	9522	Survey estimates are systematically high compared to admin estimates and estimates from other established survey series (2008 DHS, 2007 MICS).
Zambia	LSMS	1998	Central Statistical Office (Zambia), London School of Hygiene and Tropical Medicine. Zambia Living Conditions Monitoring Survey 1998. Lusaka, Zambia: Central Statistical Office (Zambia).	14015	Estimates considered implausible (zero values).
Zambia	Zambia Living Conditions Monitoring Survey	2002-2003	Central Statistical Office (Zambia). Zambia Living Conditions Monitoring Survey 2002-2003. Lusaka, Zambia: Central Statistical Office (Zambia).	14027	Estimates considered implausible (zero values).
Ethiopia	LSMS	2015-2016	Central Statistical Agency (Ethiopia), World Bank. Ethiopia Socioeconomic Survey 2015-2016. Washington DC, United States: World Bank, 2015.	286657	Estimates considered implausible (zero values).
Senegal	Multiple Indicator Cluster Survey (MICS)	2015-2016	National Agency of Statistics and Demography (Senegal), United Nations Children's Fund (UNICEF). Senegal - Dakar Urban Multiple Indicator Cluster Survey 2015-2016. New York, United States: United Nations Children's Fund (UNICEF), 2018.	287639	Estimates considered implausible (zero values).

Country	Series	Year(s)	Citation	NID♦	Rationale for exclusion
Uganda	LSMS	2010-2011	Uganda Bureau of Statistics. Uganda Living Standards Measurement Survey - Integrated Survey on Agriculture 2010-2011. Washington DC, United States: World Bank.	142934	Survey estimates are systematically high compared to estimates from other established survey series (2006 DHS, 2011 DHS, 2016 DHS)
Uganda	LSMS	2011-2012	Uganda Bureau of Statistics. Uganda Living Standards Measurement Survey - Integrated Survey on Agriculture 2010-2011. Washington DC, United States: World Bank.	142935	Survey estimates are systematically high compared to estimates from other established survey series (2006 DHS, 2011 DHS, 2016 DHS)

♦NID = Data source unique identifier in the Global Health Data Exchange (GHDx) (http://ghdx.healthdata.org/). Additional information about each data source is available via the GHDx, including information about the data provider and links to where the data can be accessed or requested (where available). NIDs can be entered in the search bar to retrieve the record for a particular source.

Supplementary Table 5. Data excluded from GBD estimates but included in geostatistical model

Country	Series	Year(s)	Citation	NID♦	Rationale for exclusion
Madagascar	Multiple Indicator Cluster Survey (MICS)	2012	National Institute of Statistics (Madagascar), United Nations Children's Fund (UNICEF). Madagascar - South Multiple Indicator Cluster Survey 2012. New York, United States: United Nations Children's Fund (UNICEF), 2015.	125594	Not nationally representative. Only sampled the south of Madagascar.

♦NID = Data source unique identifier in the Global Health Data Exchange (GHDx) (http://ghdx.healthdata.org/). Additional information about each data source is available via the GHDx, including information about the data provider and links to where the data can be accessed or requested (where available). NIDs can be entered in the search bar to retrieve the record for a particular source.

Supplementary Table 6. Data excluded from geostatistical model but included in GBD estimates

Country	Series	Year(s)	Citation	NID♦	Rationale for exclusion
Guinea-Bissau	Multiple Indicator Cluster Survey (MICS)	2000	Ministry of Health (Mali), National Institute of Statistics (INSTAT) (Mali), United Nations Children's Fund (UNICEF). Mali Multiple Indicator Cluster Survey 2009-2010. New York, United States: United Nations Children's Fund (UNICEF), 2017.	4808	Estimates considered implausible (zero values).
Kenya	Kenya - North Eastern Province Multiple Indicator Cluster Survey 2007	2007	Kenya National Bureau of Statistics, United Nations Children's Fund (UNICEF). Kenya - North Eastern Province Multiple Indicator Cluster Survey 2007. Nairobi, Kenya: Kenya National Bureau of Statistics.	155335	Estimates considered implausible (zero values).
Zambia	Zambia Living Conditions Monitoring Survey	2004-2005	Central Statistical Office (Zambia). Zambia Living Conditions Monitoring Survey 2004-2005. Lusaka, Zambia: Central Statistical Office (Zambia).	14063	Estimates considered implausible (zero values).

♦NID = Data source unique identifier in the Global Health Data Exchange (GHDx) (http://ghdx.healthdata.org/). Additional information about each data source is available via the GHDx, including information about the data provider and links to where the data can be accessed or requested (where available). NIDs can be entered in the search bar to retrieve the record for a particular source.

Supplementary Table 7. Sources for covariates used in mapping.

Covariate	Temporal resolution	Source	Reference	Supporting Rationale
Urbanicity	Annual, 2000–2016	European Commission/ GHS	Pesaresi, M. *et al.* Operating procedure for the production of the Global Human Settlement Layer from Landsat data of the epochs 1975, 1990, 2000, and 2014. JRC Technical Report EUR 27741 EN; doi: 10.2788/253582 (online) (Publications Office of the European Union, 2016).	*These covariates were included as measures or proxies for connectedness and urbanicity, as EBF is typically found to be different in urban areas compared to rural locations.*<br><br>UNICEF. Exclusive breastfeeding (<6 months) dataset. Infant and young child feeding (IYCF) data. Available at: https://data.unicef.org/resources/dataset/infant-young-child-feeding/<br><br>Global Nutrition Report. 2018 Global Nutrition Report: Shining a light to spur action on nutrition. (Development Initiatives Poverty Research Ltd., 2018)<br><br>Shirima, R., Greiner, T., Kylberg, E. & Gebre-Medhin, M. Exclusive breast-feeding is rarely practised in rural and urban Morogoro, Tanzania. Public Health Nutrition **4**, 147–154 (2001).<br><br>Shirima, R., Gebre-Medhin, M. & Greiner, T. Information and socioeconomic factors associated with early breastfeeding practices in rural and urban Morogoro, Tanzania. Acta Paediatrica **90**, 936–942 (2001).<br><br>Perez-Escamilla, R. Update on the breastfeeding situation in Africa. Nutrition Research **13**, 597–609 (1993).<br><br>*Population data:*<br>Tatem, A.J. WorldPop, open data for spatial demography. *Scientific Data* **4**, 170004 (2017).
Night-time lights	Annual, 2000–2013	NOAA DMSP	Savory, P. *et al.* Intercalibration and Gaussian Process Modeling of Nighttime Lights Imagery for Measuring Urbanization Trends in Africa 2000–2013. *Remote Sensing* **9**, (2017).	
Travel time to nearest settlement >50,000 inhabitants	Static	Malaria Atlas Project, Big Data Institute, Nuffield Department of Medicine, University of Oxford	Weiss, D. J. *et al.* A global map of travel time to cities to assess inequalities in accessibility in 2015. *Nature* **533**, 333-336 (2018).	
Population	Annual, 2000–2017	WorldPop	Lloyd, C. T., Sorichetta, A. & Tatem, A. J. High resolution global gridded data for use in population studies. *Scientific Data* **4**, sdata20171 (2017).<br><br>World Pop. Get data. Available at: http://www.worldpop.org.uk/data/get_data/ (Accessed: 24th July 2017)	

Covariate	Temporal resolution	Source	Reference	Supporting Rationale
Human Development Index	Annual, 2000–2016	Kummu *et al.* (modelled)	Kummu, M. *et al.* Gridded global datasets for Gross Domestic Product and Human Development Index over 1990-2015. *Scientific Data*, **5**:180004 (2018)	Victora, C. G. *et al.* Breastfeeding in the 21st century: epidemiology, mechanisms, and lifelong effect. *The Lancet* **387**, 475–490 (2016).
Educational attainment in women of reproductive age (15-49 years old)	Annual, 2000–2017	Institute for Health Metrics and Evaluation (IHME), University of Washington	Graetz, N. *et al.* Local variation in educational attainment in low- and middle-income countries, 2000–2017. *In review*.	Coutinho, S. B., de Lira, P.I.C., de Carvalho Lima, M. & Ashworth, A. Comparison of the effect of two systems for the promotion of exclusive breastfeeding. *The Lancet* **366**, 1094–1100 (2005).<br><br>Cernadas, J. M. C., Noceda, G., Barrera, L., Martinez, A. M. & Garsd, A. Maternal and Perinatal Factors Influencing the Duration of Exclusive Breastfeeding During the First 6 Months of Life. *J Hum Lact* **19**, 136–144 (2003).
Nutritional yield for vitamin A	Static	Herrero *et al.* (modelled)	Herrero, M. *et al.* Farming and the geography of nutrient production for human use: a transdisciplinary analysis. *Lancet Planet. Health* **1**, e33–e42 (2017).	Lartey, A. Maternal and child nutrition in Sub-Saharan Africa: challenges and interventions. *Proceedings of the Nutrition Society* **67**, 105–108 (2008).<br><br>Wray, J. D. Maternal Nutrition, Breast-Feeding and Infant Survival. in *Nutrition and Human Reproduction* (ed. Mosley, W. H.) 197–229 (Springer US, 1978). doi:10.1007/978-1-4684-0790-7_12
Human Immuno-deficiency Virus (HIV)	Annual, 2000–2017	IHME, University of Washington	Dwyer-Lindgren, L. *et al.* Mapping HIV prevalence in sub-Saharan Africa between 2000 and 2017. *Nature* (2019)	Bland, R. M. *et al.* Intervention to promote exclusive breast-feeding for the first 6 months of life in a high HIV prevalence area. *AIDS* **22**, 883 (2008).<br><br>Doherty, T. *et al.* Effectiveness of the WHO/UNICEF guidelines on infant feeding for HIV-positive women: results from a prospective cohort study in South Africa. *AIDS* **21**, 1791 (2007).

Covariate	Temporal resolution	Source	Reference	Supporting Rationale
				Thairu, L. N., Pelto, G. H., Rollins, N. C., Bland, R. M. & Ntshangase, N. Sociocultural influences on infant feeding decisions among HIV-infected women in rural Kwa-Zulu Natal, South Africa. *Maternal & Child Nutrition* **1**, 2–10 (2005).<br><br>Bland, R. M., Rollins, N. C., Coutsoudis, A. & Coovadia, H. M. Breastfeeding practices in an area of high HIV prevalence in rural South Africa. *Acta Paediatrica* **91**, 704–711 (2002).

Supplementary Table 8. Fitted model parameters

Parameter	**North Africa**			**Central sub-Saharan Africa**			**Eastern sub-Saharan Africa**			**Western sub-Saharan Africa**			**Southern sub-Saharan Africa**		
Quantile	**2.5th**	**50th**	**97.5th**	**2.5th**	**50th**	**97.5th**	**2.5th**	**50th**	**97.5th**	**2.5th**	**50th**	**97.5th**	**2.5th**	**50th**	**97.5th**
β_0	-0.1462	-0.0415	0.0629	-0.5129	-0.2069	0.0988	-0.6069	-0.3637	-0.1267	-0.4200	-0.1388	0.1421	-0.3082	-0.0069	0.2939
β_1 (GAM)	-0.0883	0.0160	0.1208	0.0646	0.1977	0.3192	-0.1143	-0.0013	0.1109	-0.4187	-0.2344	-0.0502	-0.0929	0.1263	0.3500
β_1 (BRT)	0.9300	0.1006	1.0827	0.4819	0.5662	0.6506	0.5230	0.5843	0.6456	0.4150	0.4709	0.5269	0.4513	0.5956	0.7405
β_1 (Lasso)	-0.1224	-0.0224	0.0768	0.1090	0.2356	0.3642	0.2888	0.4168	0.5357	0.5725	0.7634	0.9541	0.0282	0.2771	0.5223
Nominal range	2.7376	5.8356	12.0506	3.6090	4.7328	6.5772	4.3078	5.4416	6.6638	2.8637	3.4191	3.9838	2.9145	5.7189	10.5247
Nominal variance	0.0090	0.0290	0.0833	0.2809	0.3879	0.5591	0.2882	0.3870	0.5379	0.4892	0.6213	0.7569	0.0651	0.1564	0.3451
Precision for γ_{ci}	1574.64	14527.76	75595.18	1.8815	5.8201	20.3750	4.7134	12.6720	36.4112	1.2403	2.9760	5.7166	3.6622	15.2776	104.7565
Precision for ϵ_i	2089.64	16121.34	83742.48	1213.19	14355.96	70737.40	1438.27	14449.73	71913.64	1990.83	15633.41	178313.84	1323.07	12958.36	66808.70
AR1 ρ	0.8702	0.9795	0.997	0.8684	0.9308	0.9748	0.8836	0.9211	0.9501	0.6906	0.7530	0.7953	0.7639	0.9149	0.9723

For each modeling region, the following parameters are reported: intercept (β_0), regression coefficients (β_1) corresponding to the three sub-models (generalised additive model (GAM), boosted-regression trees (BRT), and Lasso), nominal range, nominal variance, precision for country-level random effects (γ_{ci}), precision for independent and identically distributed nugget (uncorrelated error term) effect (ϵ_i), and hyperparameter (ρ) for the temporal first-order autoregressive (AR1) covariance function at 2.5th, 50th and 97.5th quantiles.

Supplementary Table 9. In-sample and out-of-sample validation metrics from the covariate sensitivity analysis by level of aggregation.

Model	Metric	Country Level		First Administrative Level		Second Administrative Level	
		IS	OOS	IS	OOS	IS	OOS
**Covariate Sensitivity Analysis**							
**Model 1: Raw covariates**	**ME**	-0.0139	0.2407	-0.0139	0.2407	-0.0139	0.2407
	**RMSE**	7.3665	13.5072	13.1176	17.4058	17.7537	21.1484
	**Coverage (%)**	88.6509	84.3821	88.6509	84.3621	88.6509	84.3621
**Model 2: Stacking predictions as covariates**	**ME**	-0.4875	-0.0506	-0.4875	-0.0506	-0.4875	-0.0506
	**RMSE**	1.8526	14.9421	7.4135	18.6234	12.2126	22.3088
	**Coverage (%)**	95.8904	79.2453	95.8904	79.2453	95.8904	79.2453
**Model 3: Gaussian Process (GP)**	**ME**	-0.4875	-0.0506	-0.4875	-0.0506	-0.4875	-0.0506
	**RMSE**	1.8526	14.9421	7.4135	18.6234	12.2126	22.3089
	**Coverage (%)**	95.8904	79.2453	95.8904	79.2453	95.8904	79.2453
**Model 4: Raw covariates + GP**	**ME**	-0.0139	0.1267	-0.0139	0.1267	-0.0139	0.1267
	**RMSE**	0.9994	14.1258	6.2024	17.6270	12.5794	21.2935
	**Coverage (%)**	97.5589	91.4886	97.5589	91.4886	97.5589	91.4886
***Model 5: Stacking covariates + GP**	**ME**	-0.3696	0.4233	-0.3696	0.4233	-0.3696	0.4233
	**RMSE**	1.4044	12.9001	5.6345	16.5685	11.4187	20.4707
	**Coverage (%)**	97.5889	87.6537	97.5889	87.6537	97.5889	87.6537
**24-hour recall period Sensitivity Analysis**							
**Only surveys with 24-hour recall period**	**ME**	-0.3306	1.0198	-0.3306	1.0198	-0.3306	1.0198
	**RMSE**	1.2691	13.2002	5.5748	16.7282	11.4241	20.6138
	**Coverage (%)**	97.9263	87.1779	97.9263	87.1779	97.9263	87.1779
***All available surveys**	**ME**	-0.3696	0.4233	-0.3696	0.4233	-0.3696	0.4233
	**RMSE**	1.4044	12.9001	5.6345	16.5685	11.4187	20.4707
	**Coverage (%)**	97.5889	87.6537	97.5889	87.6537	97.5889	87.6537

For each model configuration, the following metrics are reported: mean error (ME (percentage points)), root-mean-square error (RMSE (percentage points)), and 95% prediction interval coverage (“coverage” (%)).
*These models were chosen as our final models based on results from sensitivity analyses.

Supplementary Table 10. List of surveys used in the geostatistical model that are not nationally representative

Country	Series	Year(s)	Citation	NID♦	Subnational region
Madagascar	Multiple Indicator Cluster Survey (MICS)	2012	National Institute of Statistics (Madagascar), United Nations Children's Fund (UNICEF). Madagascar - South Multiple Indicator Cluster Survey 2012. New York, United States: United Nations Children's Fund (UNICEF), 2015.	125594	Only sampled the south of Madagascar.
Egypt	Multiple Indicator Cluster Survey (MICS)	2013-2014	El-Zanaty and Associates, Ministry of Health and Population (Egypt), United Nations Children's Fund (UNICEF). Egypt IPHN Rural Districts Multiple Indicator Cluster Survey 2013-2014. New York, United States: United Nations Children's Fund (UNICEF), 2016.	159617	Only sampled selected disadvantaged rural districts in Upper and Lower Egypt.
Ghana	Multiple Indicator Cluster Survey (MICS)	2010-2011	Institute of Statistical, Social and Economic Research, University of Ghana, United Nations Children's Fund (UNICEF). Ghana - Accra Multiple Indicator Cluster Survey 2010-2011. New York, United States: United Nations Children's Fund (UNICEF), 2014.	56241	Only sampled 5 highly densely populated localities in Ghana.
Kenya	Multiple Indicator Cluster Survey (MICS)	2008	Kenya National Bureau of Statistics, United Nations Children's Fund (UNICEF). Kenya - Eastern Province Multiple Indicator Cluster Survey 2008. Nairobi, Kenya: Kenya National Bureau of Statistics.	7401	Only sampled Eastern Province in Kenya.
Kenya	Multiple Indicator Cluster Survey (MICS)	2009	Kenya National Bureau of Statistics, United Nations Children's Fund (UNICEF). Kenya - Coast Multiple Indicator Cluster Survey 2009. New York, United States: United Nations Children's Fund (UNICEF), 2014.	56420	Only sampled Mombasa district's informal settlements in Kenya.
Kenya	Multiple Indicator Cluster Survey (MICS)	2011	Kenya National Bureau of Statistics, United Nations Children's Fund (UNICEF). Kenya - Nyanza Province Multiple Indicator Cluster Survey 2011. Nairobi, Kenya: Kenya National Bureau of Statistics.	135416	Only sampled Nyanza Province in Kenya.

Kenya	Multiple Indicator Cluster Survey (MICS)	2013-2014	Kenya - Bungoma County Multiple Indicator Survey 2013-2014. New York, United States: United Nations Children's Fund (UNICEF), 2015.	203654 †	Only sampled Bungoma County in Kenya.
Kenya	Multiple Indicator Cluster Survey (MICS)	2013-2014	Kenya - Kakamega County Multiple Indicator Survey 2013-2014. New York, United States: United Nations Children's Fund (UNICEF), 2015.	203663 †	Only sampled Kakamega County in Kenya.
Kenya	Multiple Indicator Cluster Survey (MICS)	2013-2014	Kenya - Turkana County Multiple Indicator Survey 2013-2014. New York, United States: United Nations Children's Fund (UNICEF), 2015.	203664 †	Only sampled Turkana County in Kenya.
Somalia	Multiple Indicator Cluster Survey (MICS)	2011	Ministry of National Planning and Development (Somaliland), United Nations Children's Fund (UNICEF). Somalia - Somaliland Multiple Indicator Cluster Survey 2011. New York, United States: United Nations Children's Fund (UNICEF), 2015.	91507	Only sampled Somaliland.
Somalia	Multiple Indicator Cluster Survey (MICS)	2011	Puntland Ministry of Planning and International Cooperation (Somalia), United Nations Children's Fund (UNICEF). Somalia - Northeast Zone Multiple Indicator Cluster Survey 2011. New York, United States: United Nations Children's Fund (UNICEF), 2015.	91508	Only sampled Northeast Zone in Somalia.
South Sudan	Multiple Indicator Cluster Survey (MICS)	1999	United Nations Children's Fund (UNICEF). South Sudan Multiple Indicator Cluster Survey 1999. New York, United States: United Nations Children's Fund (UNICEF).	12232	Sampled before Sudan split.

♦NID = Data source unique identifier in the Global Health Data Exchange (GHDx) (http://ghdx.healthdata.org/). Additional information about each data source is available via the GHDx, including information about the data provider and links to where the data can be accessed or requested (where available). NIDs can be entered in the search bar to retrieve the record for a particular source.

†Data source is not publicly available due to restrictions by the data provider and was used under license for the current study.

Supplementary Table 11. National and subnational achievement of WHO GNT (2017)

WHO GNT met at national level	WHO GNT met for all first administrative units	WHO GNT met for all second administrative units
Burundi<br>Eritrea<br>Ethiopia<br>Lesotho<br>Liberia<br>Malawi<br>Rwanda<br>São Tomé and Príncipe<br>Tanzania<br>Uganda<br>Zambia	Burundi<br>Malawi<br>Rwanda<br>São Tomé and Príncipe	Malawi<br>Rwanda<br>São Tomé and Príncipe

Supplementary Table 12. National and subnational achievement of WHO GNT (2025)

WHO GNT met at national level	WHO GNT met for all first administrative units	WHO GNT met for all second administrative units
Angola<br>Burkina Faso<br>Burundi<br>Democratic Republic of the Congo<br>Eritrea<br>Ethiopia<br>Guinea-Bissau<br>Lesotho<br>Liberia<br>Malawi<br>Mauritania<br>Rwanda<br>São Tomé and Príncipe<br>Sierra Leone<br>Sudan<br>Tanzania<br>Uganda<br>Zambia	Burundi<br>Guinea-Bissau<br>Lesotho<br>Malawi<br>Rwanda<br>São Tomé and Príncipe<br>Sierra Leone<br>Zambia	Guinea-Bissau<br>Rwanda<br>São Tomé and Príncipe
