# Supplementary material for: Mapping exclusive breastfeeding in Africa between 2000 and 2017
Source: Nat Med. 2019 Jul 22;25(8):1205–12. doi: 10.1038/s41591-019-0525-0 (PMC6749549; doi:10.1038/s41591-019-0525-0)
Supplement: Supplementary file 2 — Reporting Summary [file 41591_2019_525_MOESM2_ESM.pdf]

nature research

Corresponding author(s): Simon I. Hay

Last updated by author(s): Jun 5, 2019

Reporting Summary

Nature Research wishes to improve the reproducibility of the work that we publish. This form provides structure for consistency and transparency in reporting. For further information on Nature Research policies, see Authors & Referees and the Editorial Policy Checklist.

Statistics

For all statistical analyses, confirm that the following items are present in the figure legend, table legend, main text, or Methods section.

n/a	Confirmed	
☐	☒	The exact sample size (n) for each experimental group/condition, given as a discrete number and unit of measurement
☐	☒	A statement on whether measurements were taken from distinct samples or whether the same sample was measured repeatedly
☒	☐	The statistical test(s) used AND whether they are one- or two-sided<br>*Only common tests should be described solely by name; describe more complex techniques in the Methods section.*
☐	☒	A description of all covariates tested
☐	☒	A description of any assumptions or corrections, such as tests of normality and adjustment for multiple comparisons
☐	☒	A full description of the statistical parameters including central tendency (e.g. means) or other basic estimates (e.g. regression coefficient) AND variation (e.g. standard deviation) or associated estimates of uncertainty (e.g. confidence intervals)
☒	☐	For null hypothesis testing, the test statistic (e.g. F, t, r) with confidence intervals, effect sizes, degrees of freedom and P value noted<br>*Give P values as exact values whenever suitable.*
☐	☒	For Bayesian analysis, information on the choice of priors and Markov chain Monte Carlo settings
☒	☐	For hierarchical and complex designs, identification of the appropriate level for tests and full reporting of outcomes
☒	☐	Estimates of effect sizes (e.g. Cohen's d, Pearson's r), indicating how they were calculated

Our web collection on statistics for biologists contains articles on many of the points above.

Software and code

Policy information about availability of computer code

Data collection	No primary data collection was carried out for these analyses.
Data analysis	These analyses were carried out using R version 3.5.0. The main geostatistical models were fit using R-INLA version 18.07.12. All code used for these analyses is publicly available online at https://github.com/ihmeuw/lbd/tree/ebf-africa-2019.

For manuscripts utilizing custom algorithms or software that are central to the research but not yet described in published literature, software must be made available to editors/reviewers. We strongly encourage code deposition in a community repository (e.g. GitHub). See the Nature Research guidelines for submitting code & software for further information.

Data

Policy information about availability of data

All manuscripts must include a data availability statement. This statement should provide the following information, where applicable:

- Accession codes, unique identifiers, or web links for publicly available datasets
- A list of figures that have associated raw data
- A description of any restrictions on data availability

The findings of this study are supported by data that are available in public online repositories, data that are publicly available upon request from the data provider, and data that are not publicly available due to restrictions by the data provider and which were used under license for the current study. Detailed tables of data sources can be found in Supplementary Tables 2-6. More information about each data source is available on the Global Health Data Exchange (http://ghdx.healthdata.org/), including information about the data provider and links to where the data can be accessed or requested (where available).

Administrative boundaries were retrieved from the Global Administrative Unit Layers (GAUL) dataset, implemented by FAO within the CountrySTAT and Agricultural Market Information System (AMIS) projects [52]. Land cover was retrieved from the online Data Pool, courtesy of the NASA EOSDIS Land Processes Distributed Active Archive Center (LP DAAC), USGS/Earth Resources Observation and Science (EROS) Center, Sioux Falls, South Dakota [51]. Lakes were retrieved from the Global Lakes and Wetlands Database (GLWD), courtesy of the World Wildlife Fund and the Center for Environmental Systems Research, University of Kassel [53].

Populations were retrieved from WorldPop [55].

All estimates produced as part of these analyses are publicly available from the Global Health Data Exchange (http://ghdx.healthdata.org/record/ihme-data/africa-exclusive-breastfeeding-prevalence-geospatial-estimates-2000-2017) and via a user-friendly data visualisation tool (https://vizhub.healthdata.org/lbd/ebf).

Field-specific reporting

Please select the one below that is the best fit for your research. If you are not sure, read the appropriate sections before making your selection.

☒ Life sciences ☐ Behavioural & social sciences ☐ Ecological, evolutionary & environmental sciences

For a reference copy of the document with all sections, see nature.com/documents/nr-reporting-summary-flat.pdf

Life sciences study design

All studies must disclose on these points even when the disclosure is negative.

Sample size

Sample size was calculated as the number of unique data source-location pairs with survey responses regarding the feeding of children less than 6 months old at the time of the survey, in order to estimate exclusive breastfeeding (EBF) prevalence). This sample size is reported in the methods section: "After performing the data processing described above, our final dataset consisted of 60,083 clusters (33,341 of which were GPS-located data point and 26,742 of which were polygon data) from 188 surveys (181 surveys with microdata and 7 survey reports) representing 153,465 children across 49 African countries." This is an observational study with no hypothesis testing and the sample size was not pre-specified. We evaluate the overall performance of our modelling strategy, given the available data, as part of a validation exercise as described in the 'Model validation' section of the methods, and as reported in the Supplementary Information (Supplementary Table 9).

Data exclusions

Surveys or reports that did not contain the relevant variable (i.e., survey responses regarding the feeding practices of children less than 6 months old at the time of the survey) or did not contain subnational geographic detail or could otherwise not be geolocated, or were outside the geographic (i.e., Africa) or temporal (i.e., 1998-2017) scope of the study, were excluded as not relevant for these analyses. Surveys with microdata (i.e., individual-level responses) were excluded if they did not contain questions about the age of the child, whether the child is still being breastfed, and whether the child has consumed other food or liquid items. Survey reports without microdata were excluded if the survey did not contain a prevalence number for EBF with a sample size or the lower and upper bounds for the 95% confidence interval. Additionally, we excluded surveys that only asked mothers and caregivers if infants had been exclusively breastfed (e.g., "did you exclusively breastfeed?") without ascertaining further information. This exclusion criterion was established after finding, by comparing responses in surveys containing both types of questions, that many mothers and caregivers stated infants had exclusively breastfed but also answered that they had received food or water in the 24-hour recall questions. This may be due to the respondent misunderstanding the meaning of "exclusive breastfeeding" or the question may have been misinterpreted with translation. Instead, we classified children as exclusively breastfed if survey responses indicated they received only breast-milk and medicines (i.e., oral rehydration salts, vitamins, or other medicines) without other foods or liquids on the 24-hour period prior to the survey.

Replication

This is an observational study using many years of survey and report data and in principle could be replicated. Due to the time required to extract, process, and geo-locate all data, as well as to run the statistical models, we have not undertaken an explicit replication analysis.

Randomization

Randomization was not relevant to this study. This analysis is an observational mapping study and there were no experimental groups.

Blinding

Blinding was not relevant to this study, as it was an observational study using survey and report data.

Reporting for specific materials, systems and methods

We require information from authors about some types of materials, experimental systems and methods used in many studies. Here, indicate whether each material, system or method listed is relevant to your study. If you are not sure if a list item applies to your research, read the appropriate section before selecting a response.

Materials & experimental systems

n/a	Involved in the study
☒	☐ Antibodies
☒	☐ Eukaryotic cell lines
☒	☐ Palaeontology
☒	☐ Animals and other organisms
☒	☐ Human research participants
☒	☐ Clinical data

Methods

n/a	Involved in the study
☒	☐ ChIP-seq
☒	☐ Flow cytometry
☒	☐ MRI-based neuroimaging
